# FFAR4 improves the senescence of tubular epithelial cells by AMPK/SirT3 signaling in acute kidney injury

**DOI:** 10.1038/s41392-022-01254-x

**Published:** 2022-11-30

**Authors:** Letian Yang, Bo Wang, Fan Guo, Rongshuang Huang, Yan Liang, Lingzhi Li, Sibei Tao, Ting Yin, Ping Fu, Liang Ma

**Affiliations:** 1 Kidney Research Institute, National Clinical Research Center for Geriatrics and Division of Nephrology, West China Hospital of Sichuan University, Chengdu, 610041 China; 2 Research Core Facility of West China Hospital, Chengdu, 610041 China

**Keywords:** Kidney diseases, Ageing

## Abstract

Acute kidney injury (AKI) is a serious clinical complication with high morbidity and mortality rates. Despite substantial progress in understanding the mechanism of AKI, no effective therapy is available for treatment or prevention. We previously found that G protein-coupled receptor (GPCR) family member free fatty acid receptor 4 (FFAR4) agonist TUG891 alleviated kidney dysfunction and tubular injury in AKI mice. However, the versatile role of FFAR4 in kidney has not been well characterized. In the study, the expression of FFAR4 was abnormally decreased in tubular epithelial cells (TECs) of cisplatin, cecal ligation/perforation and ischemia/reperfusion injury-induced AKI mice, respectively. Systemic and conditional TEC-specific knockout of FFAR4 aggravated renal function and pathological damage, whereas FFAR4 activation by TUG-891 alleviated the severity of disease in cisplatin-induced AKI mice. Notably, FFAR4, as a key determinant, was firstly explored to regulate cellular senescence both in injured kidneys of AKI mice and TECs, which was indicated by senescence-associated β-galactosidase (SA-β-gal) activity, marker protein p53, p21, Lamin B1, phospho-histone H2A.X, phospho-Rb expression, and secretory phenotype IL-6 level. Mechanistically, pharmacological activation and overexpression of FFAR4 reversed the decrease of aging-related SirT3 protein, where FFAR4 regulated SirT3 expression to exhibit anti-senescent effect via Gq subunit-mediated CaMKKβ/AMPK signaling in cisplatin-induced mice and TECs. These findings highlight the original role of tubular FFAR4 in cellular senescence via AMPK/SirT3 signaling and identify FFAR4 as a potential drug target against AKI.

## Introduction

AKI is a potentially life-threatening condition with a high morbidity and mortality, characterized by a reduced urine output, significant rise in serum creatinine, or both. 10-15% of admitted patients develop AKI, and its incidence is over 50% of patients in intensive care unit.^1^ The primary clinical causes of AKI consist of sepsis, and nephrotoxic medications, ischemia-reperfusion injury.^2^ About 20% of AKI cases are related to the exposure to nephrotoxic drugs.^3^ Cisplatin is a potent chemotherapeutic agent to treat various tumors, like ovarian, testicular, cervical and bladder cancers. Nevertheless, cisplatin is also notorious for the adverse effects on normal organs and tissues, specifically in kidney, restricting its usage and efficacy.^4–6^ Cisplatin accumulates in the kidney at high concentrations in the proximal tubular epithelium (about 5-times greater than in the blood), provoking inflammation, injury as well as death of tubular cells, a critical factor in determining AKI.^4–8^

In addition to direct tubular toxicity, there is increasing evidence supporting a role for cellular senescence, which has long been considered an incidental and uncontrolled form of cell fate, in the pathophysiological mechanism responsible for cisplatin-induced AKI.^9,10^ Cellular senescence is a usually irreversible and stable state of proliferative arrest associated with functional, structural and morphological variations, involving the increased secretion and expression of mediators of fibrosis, inflammation, or tissue remodeling, as senescence-associated secretory phenotype (SASP).^11,12^ Currently, factors that induce cellular senescence are dysfunction of mitochondria, DNA damage, oxidative stress, telomere shortening together with repetitive cell division, most of which initiate cell cycle arrest via the two core signaling pathways, the p16^Ink4a^ and p53/p21 pathways, which interact with each other but independently regulate the cell cycle process.^11,13^

Both the medulla and cortex of the kidney undergo senescence when AKI develops comprising vascular smooth muscle cells, TECs, endothelial cells, interstitial as well as podocytes cells, with TECs being the most frequent cells experiencing senescence and sensitive to injury.^9,10,14,15^ Senescence of renal tubular cells is an essential mechanism in the early stages that contributes to the accumulation of senescent cells after renal injury.^15,16^ A high level of SASP such as IL-6 could be determined in the acute-phase of AKI.^10,13^ A recent study found that long-term low-dose cisplatin exposure could induce premature cell senescence in kidneys of mice, which is associated with chronic kidney disease after cisplatin-induced AKI.^17^ Collectively, cell senescence is a principal element of cisplatin-induced AKI, however, the mechanisms involved remain unclear.

G protein-coupled receptors (GPCRs) participate in a variety of physiological functions, and several GPCRs play critical physiological and pathophysiological roles in kidney function.^18^ FFAR4, also known as GPR120, is a member of the GPCR family.^19^ Extensively distributed FFAR4 has been confirmed to serve a pivotal role in improving inflammation, elevating lipid metabolism and insulin sensitivity, and when activated through endogenous ligand long-chain fatty acids or synthetic agonist TUG-891.^20–25^ In our previous study, podocyte FFAR4 of the glomerulus was found to be negatively correlated with diabetic nephropathy progression, and FFAR4 activation in podocytes ameliorated kidney fibrosis and inflammation to protect against the disease.^26^ Additionally, FFAR4 agonist TUG891 could improve cisplatin-induced kidney dysfunction and injury through suppressing endoplasmic reticulum stress as well as apoptosis in renal tubular cells.^27^

However, the versatile function and mechanism of FFAR4 in kidneys has not been well described. The current study investigated the effect of tubular FFAR4 in AKI via AMPK/SirT3 signaling-mediated cell senescence and identified FFAR4 as a promising drug target against AKI.

## Results

### Activation of FFAR4 by agonist TUG891 alleviated AKI, while FFAR4 deficiency aggravated the severity of disease

**Fig. 1:**
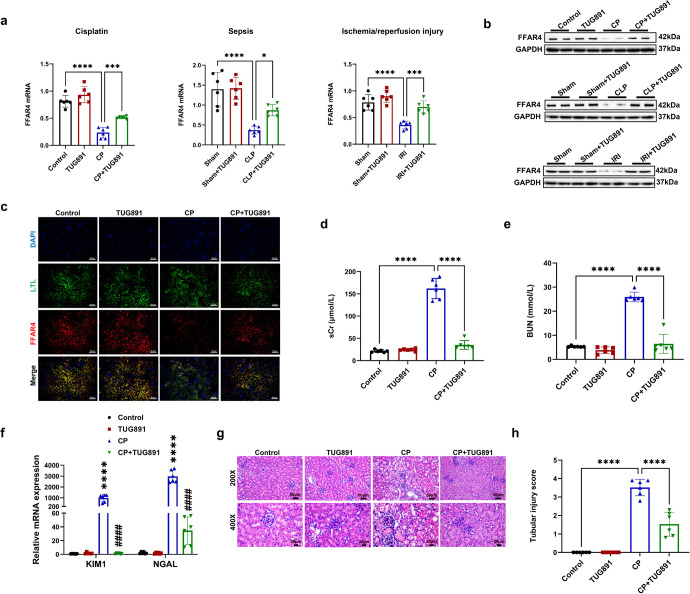
Activation of free fatty acid receptor 4 (FFAR4) by agonist TUG891 alleviated acute kidney injury (AKI) in mice. **a** Relative mRNA expression of FFAR4 in kidney tissues (*n* = 6). **b** Protein expression of FFAR4 in kidney tissues (*n* = 6). **c** Immunofluorescence of FFAR4 (red) in proximal tubules (LTL, green) in kidney sections (200×, scale bar = 50 μm). **d** Serum creatinine (sCr) in mice (*n* = 6). **e** Blood urea nitrogen (BUN) in mice (*n* = 6). **f** Relative mRNA expression of NGAL and KIM1 in kidney tissues (*n* = 6; ****P < 0.0001, CP vs. Control; ^####^*P* < 0.0001, CP + TUG891 vs. CP). **g** Representative images of hematoxylin and eosin (H&E) staining (200×, scale bar = 50 μm; 400×, scale bar = 20 μm). **h** Tubular injury scores of kidney tissues (*n* = 6). Data are presented as mean ± SD. CP cisplatin. CLP cecal ligation/perforation. IRI, ischemia/reperfusion injury. ****P < 0.0001, ***P < 0.001, *P < 0.05

**Fig. 2:**
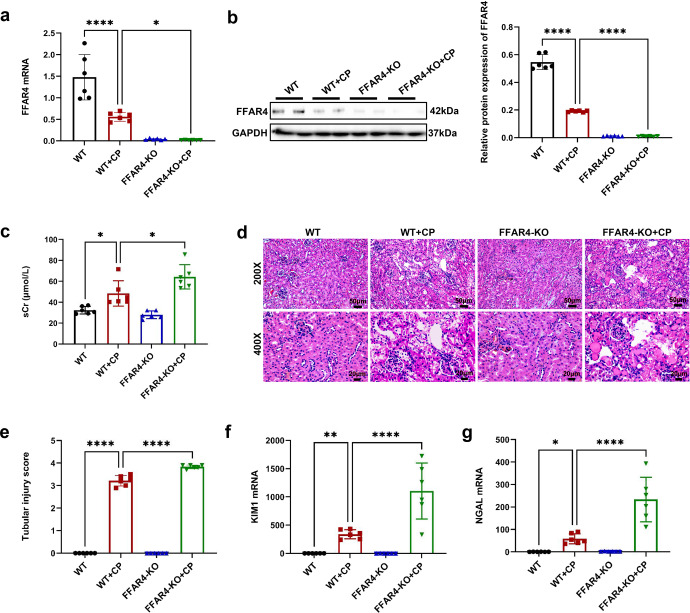
FFAR4 deficiency aggravated cisplatin-induced AKI in mice. **a** Relative mRNA expression of FFAR4 in kidney tissues (*n* = 6). **b** Protein expression of FFAR4 in kidney tissues detected by western blotting and quantified by densitometry (*n* = 6). **c** The sCr level in different groups of mice (*n* = 6). **d** Representative images of H&E staining (200×, scale bar = 50 μm; 400×, scale bar = 20 μm). **e** Tubular injury scores of kidney tissues (*n* = 6). **f** Relative mRNA expression of KIM1 in kidney tissues (*n* = 6). **g** Relative mRNA expression of NGAL in kidney tissues (*n* = 6). Data are presented as mean ± SD. CP, cisplatin. ****P < 0.0001, **P < 0.01, *P < 0.05

**Fig. 3:**
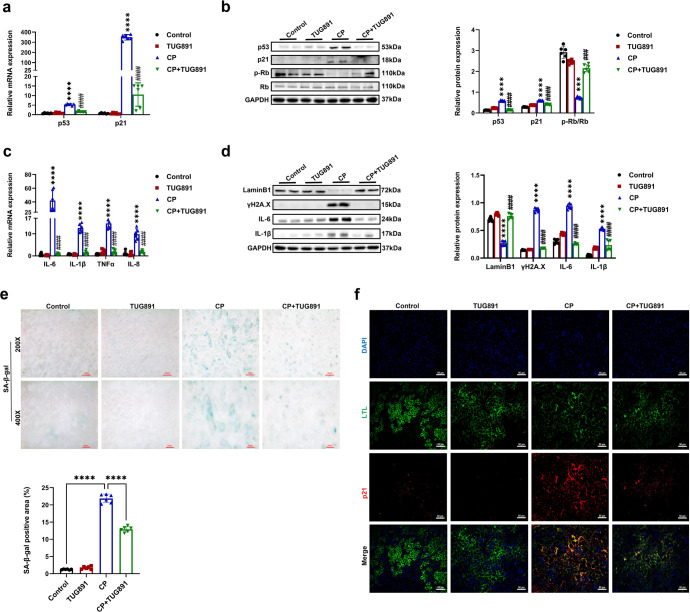
Activation of FFAR4 by TUG891 ameliorated cellular senescence in cisplatin-induced AKI mice. **a** Relative mRNA expression of p53 and p21 in kidney tissues (*n* = 6; ****P < 0.0001, CP *vs*. Control; ^####^*P* < 0.0001, CP + TUG891 vs. CP). **b** Protein expression of p53, p21, and p-Rb/Rb in kidneys was detected by western blotting and quantified by densitometry (*n* = 6; ****P < 0.0001, ***P < 0.001, CP vs. Control; ^####^*P* < 0.0001, CP + TUG891 *vs*. CP). **c** Relative mRNA expression of IL-6, IL-1β, TNF-α, and IL-8 in kidney tissues (*n* = 6; ****P < 0.0001, CP vs. Control; ^####^*P* < 0.0001, CP + TUG891 vs. CP). **d** Protein expression of LaminB1, ɣH2A.X, IL-6, and IL-1β in kidneys was detected by western blotting and quantified by densitometry (*n* = 6; ****P < 0.0001, CP vs. Control; ^####^*P* < 0.0001, CP + TUG891 *vs*. CP). **e** Representative images and quantitative analysis of SA-β-gal staining of kidney sections (200×, scale bar = 50 μm; 400×, scale bar = 20 μm; *n* = 6; ****P < 0.0001). **f** Immunofluorescence of p21 (red) in proximal tubules (LTL, green) in kidney sections (200×, scale bar = 50 μm). Data are presented as mean ± SD. CP, cisplatin

To verify the reno-protective role of FFAR4, three experimental AKI models of cisplatin (CP), cecal ligation/perforation (CLP) and ischemia/reperfusion injury (IRI) were used. In the study, TUG-891 upregulated the FFAR4 mRNA and protein expression in cisplatin, sepsis, and IRI-induced injury kidneys, respectively (Fig. 1a, b). And immunofluorescence staining displayed that the expression of FFAR4 was evidently reduced in the proximal tubules in kidney sections from cisplatin mice (Fig. 1c). Besides, by consulting public single-cell RNA sequencing database, FFAR4 was expressed abundantly in the S2 and S3 segments in proximal tubules (Supplementary Fig. 1a–f). As presented in Fig. 1d, e, g, 2a, b, d, and 3a, b, d, TUG891 treatment significantly restored the blood urea nitrogen (BUN) together with serum creatinine (sCr) levels in AKI mice, and improvement of renal tubular injury (involving necrosis, swelling, plaster composition together with inflammatory cell infiltration of tubular epithelial cells) in the AKI mice. A semi-quantitative scale of tubular injury score also confirmed that FFAR4 agonist mitigated tubular pathological changes with H&E staining (Fig. 1h, Supplementary Figs. 2c, 3c). Moreover, TUG-891 greatly decreased the mRNA levels of Lcn2 (neutrophil gelatinase-associated lipocalin, NGAL) and Havcr1 (kidney injury molecule 1, KIM1) in AKI mice induced by cisplatin (Fig. 1f).

As expected, in comparison with those of the wild-type (WT) mice, FFAR4 deficiency aggravated cisplatin, CLP, and IRI-induced kidney injury in FFAR4 knockout mice (FFAR4-KO, Fig. 2a, b, Supplementary Fig. 4), as exhibited with the raised mortality of cisplatin-treated mice (Supplementary Fig. 5) and sCr level (Fig. 2c, Supplementary Fig. 6a–7a), and more severe renal pathological injury (Fig. 2d, e, Supplementary Fig. 6–7b, c). FFAR4 gene knockout also deteriorated the KIM1 and NGAL mRNA expression in the kidneys of AKI mice induced with cisplatin (Fig. 2f, g).

### FFAR4 regulated cellular senescence in kidneys of AKI mice

Cellular senescence has been confirmed to be involved in AKI, and IL-6, p21, and SA-β-gal are extensively applied for determining senescent cells.^11–13^ During the aging or senescence process, p53 and p21 level increase, and retinoblastoma (Rb) phosphorylation expression decreases. Moreover, phosphorylated histone H2A.X (γH2A.X) is a representative marker of DNA damage, and Lamin B1 expression is decreased in senescent cells, correlated with the disruption of nuclear membrane.^11,12^ In our study, cisplatin increased protein and mRNA levels of p21 and p53, and reduced phosphorylated Rb (p-Rb) and Lamin B1 expression in mouse kidney (Fig. 3a, b, and d). Cisplatin also enhanced kidney γH2A.X expression and SASP (IL-6, IL-1β, IL-8, TNFα) mRNA level in mice (Fig. 3c, d). In addition, cisplatin triggered the number of p21-positive cells and SA-β-gal-positive senescent cells in the injured proximal tubules (Fig. 3e, f). Ki-67 is a nuclear non-histone protein rarely expressed in senescent cells, and it was observed that there was a reduction in the number of Ki-67-positive cells in kidneys of cisplatin mice (Supplementary Fig. 8). Importantly, these abnormalities in cisplatin-injured kidneys were significantly attenuated by FFAR4 agonist TUG891 treatment (Fig. 3a, f). Similarly, the SA-β-gal positive areas were raised in kidneys of AKI mice induced by IRI and CLP, and reduced following the treatment of TUG891 (Supplementary Figs. 9, 10).

**Fig. 4:**
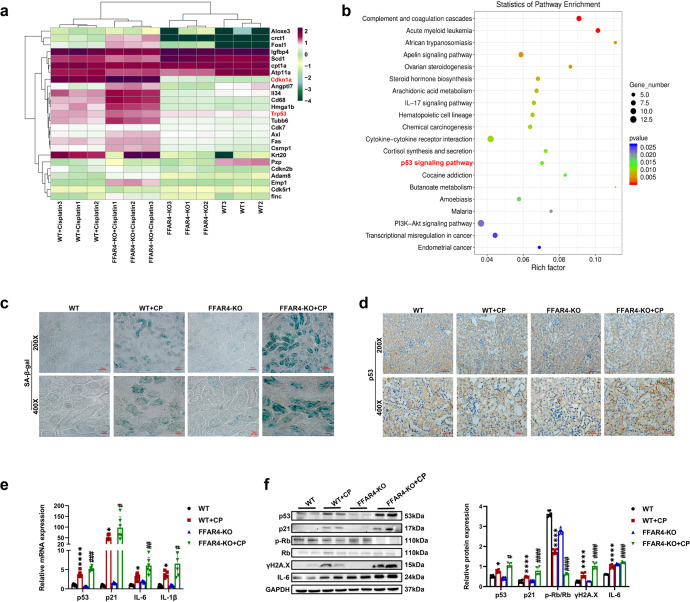
FFAR4 deficiency aggravated cellular senescence in cisplatin-induced AKI mice. **a** Representative heatmap of differentially expressed genes in the kidneys of cisplatin-induced AKI mice (*n* = 3). **b** Comparable analysis between WT + cisplatin and FFAR4-KO + cisplatin group using KEGG database. **c** SA-β-gal staining of kidney sections (200×, scale bar = 50 μm; 400×, scale bar = 20 μm). **d** Immunochemistry staining of p53 in kidney tissues (200×, scale bar = 50 μm; 400×, scale bar = 20 μm). **e** Relative mRNA expression of p53, p21, IL-6, and IL-1β in kidney tissues (*n* = 6). **f** Protein expression of p53, p21, p-Rb/Rb, ɣH2A.X, and IL-6 in kidney tissues was detected by western blotting and quantified by densitometry (*n* = 6). Data are presented as mean ± SD. CP, cisplatin. ****P < 0.0001, *P < 0.05. WT + CP vs. WT; ^####^*P* < 0.0001, ^###^*P* < 0.001, ^##^*P* < 0.01, ^#^*P* < 0.5, FFAR4-KO + CP vs. WT + CP

As expected, genetic inhibition of FFAR4 exacerbated cellular senescence in the injured kidneys of AKI mice. As displayed in Fig. 4a, b, the analysis of kidney transcriptomics indicated that gene expression associated with p53 signaling pathway, cell cycle arrest, inflammation in kidneys of FFAR4-KO mice treated through cisplatin was distinctly different from that of WT mice with cisplatin treatment. Cisplatin-induced a more severe cellular senescence phenotype by p53 positive together with SA-β-gal positive areas in the injured kidneys of FFAR4-KO mice (Fig. 4c, d, and Supplementary Fig. 11a, b). Additionally, in CLP- and IRI-induced AKI mice, in comparison to the WT group, the SA-β-gal positive regions were also elevated in the FFAR4-KO group (Supplementary Figs. 12,13). Consequentially, FFAR4 deficiency led to enhanced cellular senescence, which was demonstrated in the up-regulated protein and mRNA expression of p21 and p53, downregulation of p-Rb, and increased expression of γH2A.X and SASP (IL6, IL-1β) (Fig. 4e, f).

### Activation of FFAR4 improved cellular senescence while genetic inhibition aggravated the phenotype of cisplatin-stimulated renal tubular epithelial cells

For identifying whether cisplatin led to the in vitro cellular senescence, mouse tubular epithelial TCMK-1 cell line was used. As delineated in Supplementary Figs. 14, 15, cisplatin-induced senescence (assessed by p53, p21, and p-Rb/Rb protein expression) and downregulation of FFAR4 expression occurred in a time- and dose-dependent way. Finally, TCMK-1 cells were stimulated with 2 μg/ml cisplatin for six hours and subsequently cultured in complete medium for 24 hours.

**Fig. 5:**
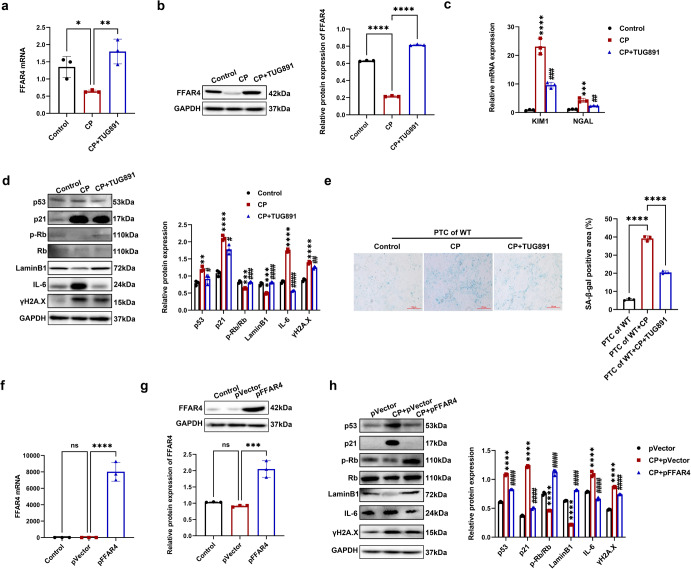
Activation of FFAR4 improved cellular senescence in cisplatin-stimulated renal tubular epithelial cells. **a**, **b** Relative mRNA and protein expression of FFAR4 in TCMK-1 cells (*n* = 3; ****P < 0.0001, **P < 0.01, *P < 0.05). **c** Relative mRNA expression of KIM1 and NGAL in TCMK-1 cells (*n* = 3; ****P < 0.0001, ***P < 0.001, CP vs. Control; ^###^*P* < 0.001, ^##^*P* < 0.01, CP + TUG891 vs. CP). **d** Protein expression of p53, p21, p-Rb/Rb, LaminB1, ɣH2A.X and IL-6 in TCMK-1 cells detected by western blotting and quantified by densitometry (*n* = 3; ****P < 0.0001, ***P < 0.001, **P < 0.01, CP vs. Control; ^####^*P* < 0.0001, ^###^*P* < 0.001, ^##^*P* < 0.01, ^#^*P* < 0.5, CP + TUG891 vs. CP). **e** Representative images and quantitative analysis of the SA-β-gal staining of renal primary tubular cells (PTCs) from WT mice (100×, scale bar = 250 μm; *n* = 3; ****P < 0.0001). **f**, **g** Relative mRNA and protein expression of FFAR4 in TCMK-1 cells transfected with empty plasmid (pVector) or plasmid expressing FFAR4 (pFFAR4) (*n* = 3; ***P < 0.001, ****P < 0.0001, ns no significant). **h** Protein expression of p53, p21, LaminB1, p-Rb/Rb, ɣH2A.X and IL-6 in TCMK-1 cells transfected with pFFAR4 detected by western blotting and quantified by densitometry (*n* = 3; ****P < 0.0001, CP + pVector *vs*. pVector; ^####^*P* < 0.0001, CP + pFFAR4 *vs*. CP + pVector). Data are presented as mean ± SD. CP cisplatin

Next, we explored whether FFAR4 activation by TUG891 or FFAR4 plasmid transfection improved senescence in TCMK-1 cells stimulated with cisplatin. As displayed in Fig. 5a, b, the FFAR4 expression markedly attenuated, and TUG-891 restored this phenomenon in TCMK-1 cells stimulated by cisplatin. In comparison with cisplatin group, TUG891 treatment markedly inhibited the relative mRNA expression of NGAL together with KIM1 in the TCMK-1 cells (Fig. 5c). Additionally, in agreement with the in vivo findings, TUG891 also downregulated p53, p21, ɣH2A.X, IL-6, as well as upregulated Lamin B1 and p-Rb in TCMK-1 cells stimulated by cisplatin (Fig. 5d). In the meantime, we extracted and cultured renal primary tubular cells (PTCs) from FFAR4 wild-type mice and observed an enhancement in the SA-β-gal positive regions in cisplatin group, which was apparently diminished after the treatment with TUG891 (Fig. 5e). Similar to the abovementioned results using TCMK-1 cell line, cisplatin raised the p21, p53, IL-6, and ɣH2A.X expression, and decreased the p-Rb and Lamin B1 expression in PTCs, where significant improvement was observed after TUG891 treatment (Supplementary Fig. 16). Furthermore, the transfection of TCMK-1 cells with plasmids for inducing the overexpression of FFAR4 (Fig. 5f, g). FFAR4 overexpression decreased the level of p53, p21, γH2A.X, and IL-6 proteins and remarkably upregulated the expression of LaminB1 and p-Rb (Fig. 5h).

**Fig. 6:**
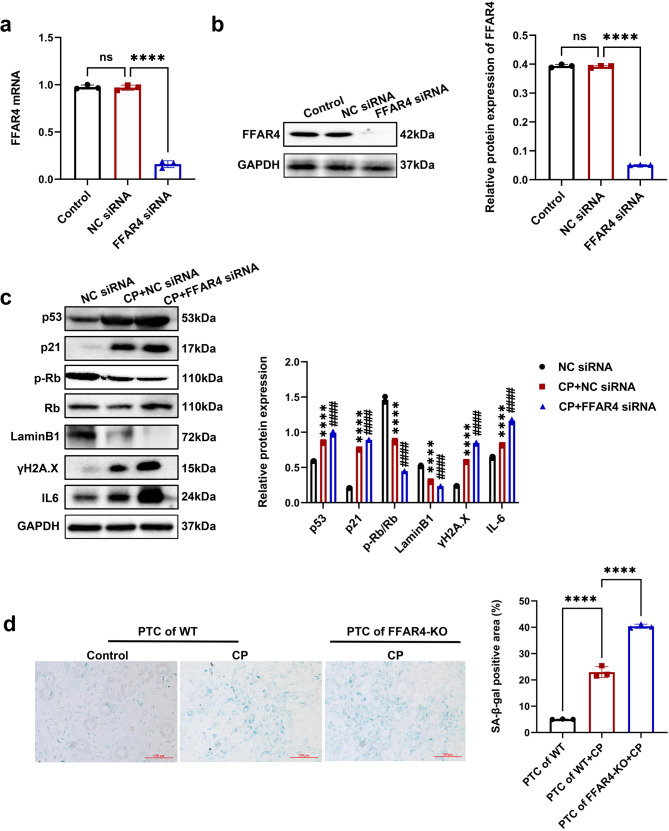
Inhibition of FFAR4 aggravated cellular senescence in cisplatin-stimulated renal tubular epithelial cells. **a**, **b** TCMK-1 cells were transfected with negative control (NC) siRNA or FFAR4 siRNA for 24 h and then treated with 2 μg/ml cisplatin for 6 h. The knockdown efficiency of FFAR4 siRNA in TCMK-1 cells was evaluated by quantitative real-time PCR analysis and western blot analysis (*n* = 3; ****P < 0.0001, ^ns^ no significant). **c** Protein expression of p53, p21, LaminB1, p-Rb/Rb, ɣH2A.X and IL-6 detected by western blotting and quantified by densitometry (*n* = 3; ****P < 0.0001, CP + NC siRNA *vs*. NC siRNA; ^####^*P* < 0.0001, CP + FFAR4 siRNA vs. CP + NC siRNA). **d** Representative images and quantitative analysis of SA-β-gal staining of renal primary tubular cells (PTCs) from WT and FFAR4-KO mice (100×, scale bar = 250 μm) (*n* = 3; ****P < 0.0001). Data are presented as mean ± SD. CP cisplatin

In another aspect, the transfection of TCMK-1 cells with siRNA was conducted for inhibiting the expression of FFAR (Fig. 6a, b). FFAR4 knockdown aggravated cisplatin-stimulated cellular senescence, evidenced by the elevated level of p53, p21, ɣH2A.X, IL-6, and the decreased level of LaminB1 and p-Rb (Fig. 6c). Compared with PTCs from wild-type mice, cisplatin-triggered SA-β-gal positive senescent cells were raised in PTCs from FFAR4-KO mice (Fig. 6d).

### FFAR4 regulated cisplatin-induced cell senescence via SirT3 activation in AKI and tubular epithelial TCMK-1 cells

**Fig. 7:**
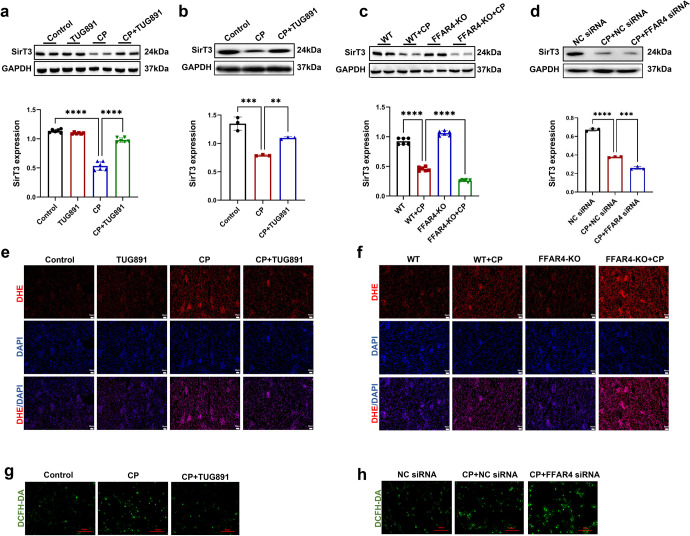
Activation of FFAR4 reversed the decrease of SirT3 in cisplatin-induced AKI. **a**, **c** Protein expression of SirT3 in kidney tissues detected by western blotting and quantified by densitometry (*n* = 6; ****P < 0.0001, *P < 0.05). **b**, **d** Protein expression of SirT3 in TCMK-1 cells detected by western blotting and quantified by densitometry (*n* = 3; ****P < 0.0001, ***P < 0.001, **P < 0.01). **e**, **f** The DHE staining of kidney sections (200×, scale bar **=** 50 μm; 400×, scale bar = 20 μm). **g**, **h** The ROS production in TCMK-1 cells was assessed by DCFH**-**DA staining (100×, scale bar = 250 μm). Data are presented as mean ± SD. CP cisplatin

SirT3 is an NAD^+^-dependent deacetylase associated with a widespread range of pathological and physiological procedures, involving both aging together with aging-associated diseases.^28^ Previous studies have indicated that SirT3 depletion caused heterochromatin loss, impaired nuclear integrity, and an acceleration of senescence in human MSCs.^29–31^ In this research, we examined the role of SirT3 in cisplatin-induced tubular cell senescence. Firstly, we observed that cisplatin-induced decrease of SirT3 expression occurred in TCMK-1 cells both in a time- and dose-dependent way (Supplementary Fig. 17). Western blot assay exhibited that the reduced protein levels of SirT3 in kidney and TCMK-1 cells injured by cisplatin were reversed with TUG891 treatment (Fig. 7a, b) and FFAR4 overexpression, respectively (Supplementary Fig. 18). Simultaneously, genetic knockout and siRNA knockdown of FFAR4 declined expression of SirT3 in TCMK-1 cells and kidney treated with cisplatin compared to the corresponding cisplatin control, respectively (Fig. 7c, d).

Existing evidence supports the involvement of ROS in p53-mediated cellular senescence.^31^ SirT3 is a known ROS inhibitor.^30,31^ Based on these evidence, we assessed ROS activity using DHE staining. As reflected in Fig. 7e, f, in AKI mice induced with cisplatin, the renal ROS content was remarkably raised, whereas it was reversed by TUG891 treatment and exacerbated by FFAR4 deficiency, respectively. Furthermore, the cisplatin-stimulated ROS level was substantially reduced by FFAR4 activation (Fig. 7g) and increased by FFAR4 silencing in TCMK-1 cells (Fig. 7h).

**Fig. 8:**
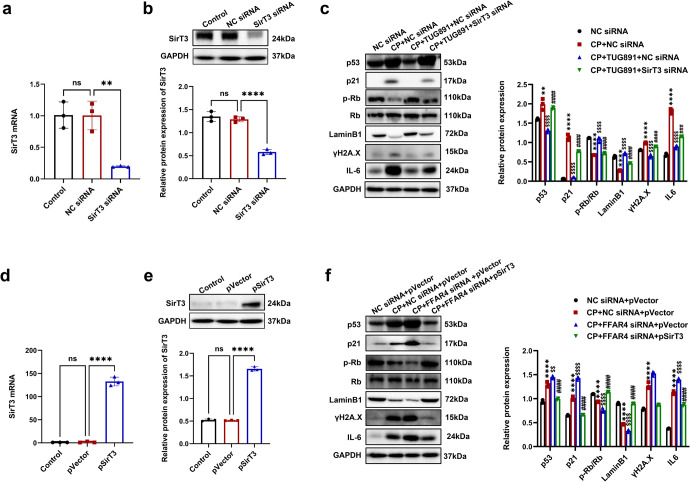
FFAR4 suppressing cisplatin-induced senescence via activating SirT3 in AKI mice. **a**, **b** Relative mRNA and protein expression of SirT3 in TCMK-1 cells transfected with SirT3 siRNA (*n* = 3; ****P < 0.001, *P < 0.05, ns. no significant). **c** Protein expression of p53, p21, LaminB1, p-Rb/Rb, ɣH2A.X and IL-6 in TCMK-1 cells transfected with SirT3 siRNA detected by western blotting and quantified by densitometry (*n* = 3; ****P < 0.0001, **P < 0.01, CP + NC siRNA *vs*. NC siRNA; ^$$$$^*P* < 0.0001, CP + TUG891 + NC siRNA *vs*. CP + NC siRNA; ^####^*P* < 0.0001, ^###^*P* < 0.001, CP + TUG891 + FFAR4 siRNA vs. CP + TUG891 + NC siRNA). **d**, **e** Relative mRNA and protein expression of SirT3 in TCMK-1 cells transfected with transfected with empty plasmid (pVector) or plasmid expressing SirT3 (pSirT3) (*n* = 3; ****P < 0.001, ns no significant). **f** Protein expression of p53, p21, LaminB1, p-Rb/Rb,ɣH2A.X and IL-6 in TCMK-1 cells trans**f**ected with empty plasmid (pVector) or plasmid expressing SirT3 (pSirT3) detected by western blotting and quantified by densitometry (*n* = 3; ****P < 0.0001, CP + NC siRNA+pVector vs. NC siRNA+ pVector; ^$$$$^*P* < 0.0001, ^$$^*P* < 0.01, CP + FFAR4 siRNA+pVector vs. CP + NC siRNA+pVector; ^####^*P* < 0.0001, CP + FFAR4 siRNA+pSirT3 vs. CP + FFAR4 siRNA+pVector). Data are presented as mean ± SD. CP cisplatin

To examine whether the anti-senescent action of FFAR4 occurs through SirT3 activation, transfection of TCMK-1 cells with SirT3 siRNA was performed (Fig. 8a, b). Notably, the SirT3 knockdown reversed anti-senescent effect of TUG891, as evidenced by the increased p53, p21, γH2A.X, IL-6, and the decreased Lamin B1, p-Rb (Fig. 8c). Moreover, SirT3 silencing promoted the intracellular ROS production (Supplementary Fig. 19). Furthermore, overexpression of SirT3 by plasmid transfection in TCMK-1 cells was investigated (Fig. 8d, e). The transfection of TCMK-1 cells was performed using SirT3 overexpression plasmid and FFAR4 siRNA. After cisplatin stimulation, the senescence phenotype was more marked in FFAR4 siRNA group, which was reversed by transfection with SirT3 overexpression plasmid. (Fig. 8f). Collectively, these data suggested that FFAR4 improved tubular senescence via the activation of SirT3 in AKI.

### FFAR4 upregulated Sirt3 expression via Gq/CaMKKβ/AMPK signaling in cisplatin-induced TCMK-1 cells and AKI mice

Considerable evidence has revealed that FFAR4 activation promoted Gαq protein- and arrestin-dependent signaling.^21,24^ The Gαq protein has been widely accepted as the upstream of phospholipase C activation, which increases intracellular 1,4,5-trisphosphate (IP3) concentration and calcium release via the IP3 receptor in the endoplasmic reticulum. When intracellular calcium level rise, CaMKKβ is activated, which ultimately phosphorylates AMPK at threonine 172.^32,33^ Recently, many studies have indicated that AMPK is an upstream signal of SirT3.^34–36^

**Fig. 9:**
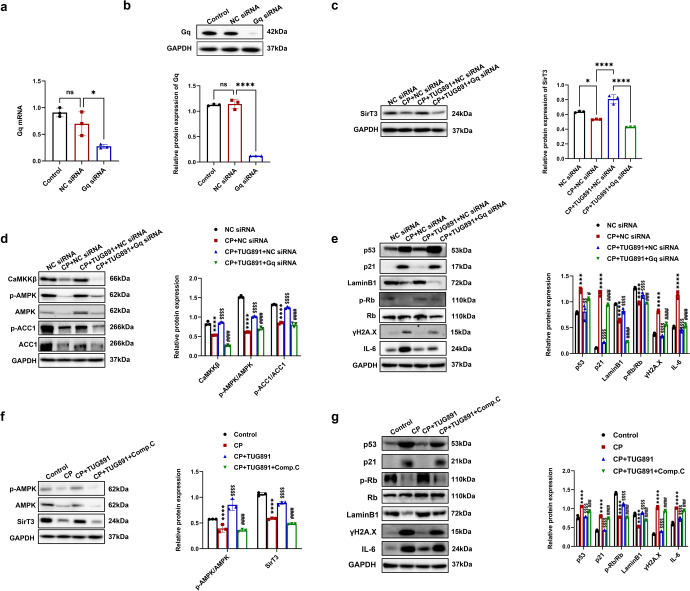
Activation of FFAR4 reversed the Sirt3 expression via Gq/CaMKKβ/AMPK signaling in cisplatin-stimulated TCMK-1 cells. **a**, **b** Relative mRNA and protein expression of Gq in TCMK-1 cells transfected with Gq siRNA (*n* = 3; *P < 0.05, ****P < 0.0001, ns no significant difference). **c** Protein expression of SirT3 in TCMK-1 cells transfected with Gq siRNA detected by western blotting and quantified by densitometry (*n* = 3; ****P < 0.0001, *P < 0.05). **d** Protein expression of CaMKKβ, p-AMPK/AMPK, and p-ACC1/ACC1 in TCMK-1 cells transfected with Gq siRNA detected by western blotting and quantified by densitometry (*n* = 3; ****P < 0.0001, CP + NC siRNA vs. NC siRNA; ^$$$$^*P* < 0.0001, CP + TUG891 + NC siRNA vs. CP + NC siRNA; ^####^*P* < 0.0001, CP + TUG891 + Gq siRNA vs. CP + TUG891 + NC siRNA). **e** Protein expression of p53, p21, p-Rb/Rb, LaminB1, ɣH2A.X and IL-6 in TCMK-1 cells transfected with Gq siRNA detected by western blotting and quantified by densitometry (*n* = 3; ****P < 0.0001, ***P < 0.001, CP + NC siRNA vs. NC siRNA; ^$$$$^*P* < 0.0001, ^$$^*P* < 0.01, CP + TUG891 + NC siRNA vs. CP + NC siRNA; ^####^*P* < 0.0001, ^#^*P* < 0.05, CP + TUG891 + Gq siRNA vs. CP + TUG891 + NC siRNA^).^ **f** TCMK-1 cells were preincubated with 10 μM compound C and TUG891 for 1 h, followed by cisplatin for 6 h. Protein expression of p-AMPK/AMPK and SirT3 detected by western blotting and quantified by densitometry (*n* = 3; ****P < 0.0001, CP vs. Control; ^$$$$^*P* < 0.0001, CP + TUG891 vs. CP; ^####^*P* < 0.0001, CP + TUG891 + Comp.C vs. CP + TUG891). **g** Protein expression of p53, p21, p-Rb/Rb, LaminB1, ɣH2A.X and IL-6 detected by western blotting and quantified by densitometry (*n* = 3; ****P < 0.0001, CP vs. Control; ^$$$$^*P* < 0.0001, CP + TUG891 vs. CP; ^####^*P* < 0.0001, CP + TUG891 + Comp.C vs. CP + TUG891). Data are presented as mean ± SD. CP cisplatin

To investigate whether FFAR4 upregulates SirT3 expression via Gq/CaMKKβ/AMPK signaling pathway, TCMK-1 cells were transfected with Gq siRNA (Fig. 9a, b). As presented in Fig. 9c, d, the upregulation of SirT3 by TUG891 was reversed by Gq silencing, and cisplatin stimulation dramatically reduced the protein level of CaMKKβ, AMPK, and ACC1. Notably, TUG891 treatment increased the p-ACC1/ACC1, p-AMPK/AMPK, and CaMKKβ expression, which was suppressed by Gq siRNA (Fig. 9d). As expected, the anti-senescent action of TUG891 in TCMK-1 cells stimulated with cisplatin was also counteracted by Gq siRNA, as indicated by the increased level of p53, p21, γH2A.X, IL-6, as well as the decreased LaminB1 and p-Rb expression (Fig. 9e). Furthermore, compound C was used to inhibit AMPK phosphorylation. Compound C could reverse FFAR4 activation-mediated SirT3 upregulation in cisplatin-stimulated TCMK-1 cells (Fig. 9f) and impaired the anti-senescent effect of TUG891 (Fig. 9g).

**Fig. 10:**
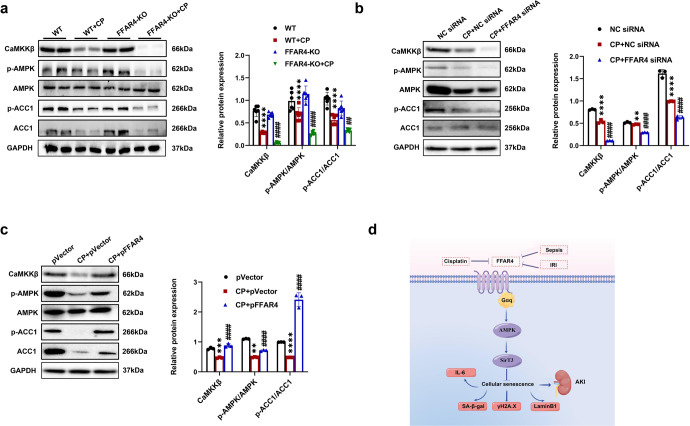
FFAR4 activates the Gq/CaMKKβ/AMPK signaling pathway in cisplatin-induced AKI and TCMK-1 cells. **a** Protein expression of CaMKKβ, p-AMPK/AMPK, and p-ACC1/ACC1 in kidneys detected by western blotting and quantified by densitometry (*n* = 6; ****P < 0.0001, WT + CP *vs*. WT; ^####^*P* < 0.0001^, ##^*P* < 0.01, FFAR4-KO + CP vs. WT + CP). **b** Protein expression of CaMKKβ, p-AMPK/AMPK, and p-ACC1/ACC1 in TCMK-1 cells transfected with FFAR4 siRNA detected by western blotting and quantified by densitometry (*n* = 3; ****P < 0.0001, **P < 0.001, CP + NC siRNA vs. NC siRNA; ^####^*P* < 0.0001, CP + FFAR4siRNA vs. CP + NC siRNA). **c** Protein expression of CaMKKβ, p-AMPK/AMPK, and p-ACC1/ACC1 in TCMK-1 cells transfected with pVector or pFFAR4 detected by western blotting and quantified by densitometry (*n* = 3; ****P < 0.0001, ***P < 0.001, **P < 0.01, CP + pVector vs. pVector; ^####^*P* < 0.0001, CP + pFFAR4 vs. CP + pVector). **d** Schematic illustration of FFAR4 in cellular senescence of AKI. Data are presented as mean ± SD. CP cisplatin

As exhibited in Fig. 10a–c, cisplatin exposure significantly decreased the CaMKKβ and AMPK phosphorylation. Compared to the corresponding cisplatin control, the p-ACC1/ACC1, p-AMPK/AMPK, and CaMKKβ level was lower in cisplatin-induced FFAR4 knockout mice (Fig. 10a), and FFAR4 knockdown in cisplatin-triggered TCMK-1 cells (Fig. 10b). Moreover, TUG891 treatment and FFAR4 overexpression could increase the p-ACC1/ACC1 ratio, p-AMPK/AMPK ratio, and CaMKKβ level in the kidneys, PTCs or TCMK-1 cells (Fig. 10c, Supplementary Figs. 20, 21).

In addition, we examined whether senescence inhibition served a protective role against cisplatin-induced AKI. Rapamycin, a suppressor of mTOR, has been revealed to suppress cellular senescence in vitro and extend the lifespan of several species.^37^ We found that rapamycin significantly improved cellular senescence in cisplatin-injured kidneys (Supplementary Fig. 22) and decreased sCr, BUN level as well as improved renal tubular injury (Supplementary Fig. 23a–d).

### Tubular epithelial cell-specific deletion of FFAR4 exacerbate kidney injury and cellular senescence in cisplatin-induced AKI mice

**Fig. 11:**
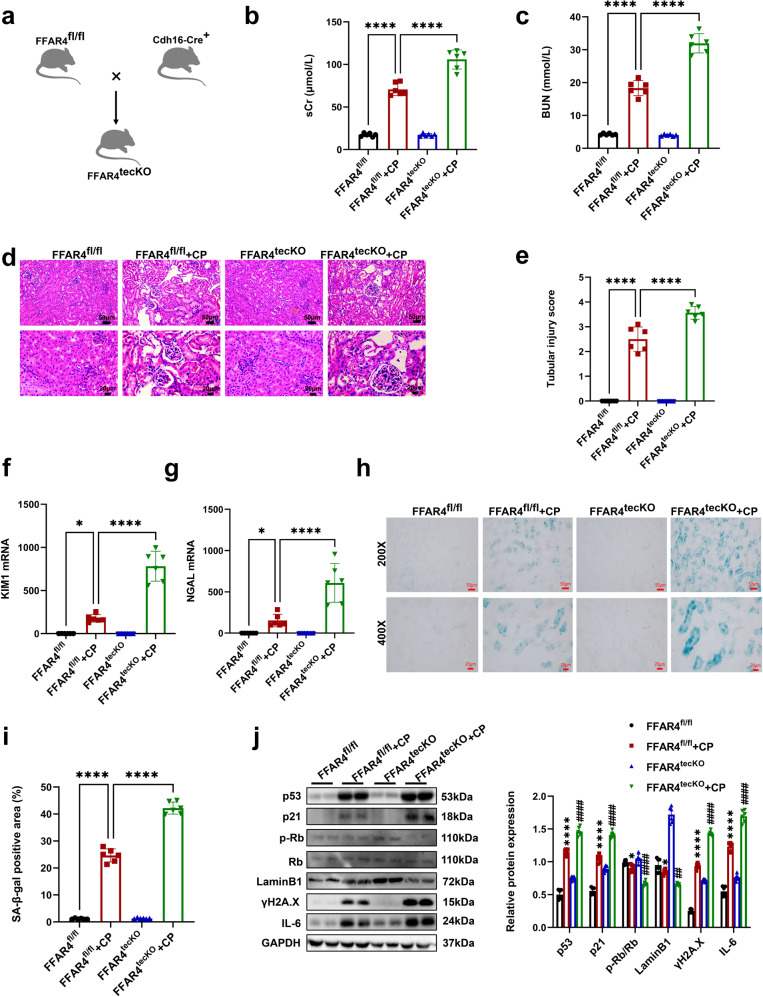
Tubular epithelial cell-specific deletion of FFAR4 aggravated kidney damage and cellular senescence in cisplatin-induced AKI mice. **a** Mating strategy to generate FFAR4 conditional KO in mouse TECs. **b**, **c** The sCr and BUN levels in different groups of mice (*n* = 6; ****P < 0.0001). **d** Representative images of H&E staining (200×, scale bar = 50 μm; 400×, scale bar = 20 μm). **e** Tubular injury scores of kidney tissues (*n* = 6; ****P < 0.0001, ***P < 0.001). **f**, **g** Relative mRNA expression of KIM1 and NGAL in kidney tissues (*n* = 6; ****P < 0.0001, *P < 0.05). **h**, **i** Representative images and quantitative analysis of SA-β-gal staining of kidney sections (200×, scale bar = 50 μm; 400×, scale bar = 20 μm; *n* = 6; ****P < 0.0001). **j** Protein expression of p53, p21, p-Rb/Rb, LaminB1, ɣH2A.X, and IL-6 in kidney tissues was detected by western blotting and quantified by densitometry (*n* = 6; ****P < 0.0001, *P < 0.05, FFAR4^fl/fl^ + CP vs. FFAR4^fl/fl^; ^####^*P* < 0.0001, ^##^*P* < 0.01, ^#^*P* < 0.05, FFAR4^tecKO^ + CP vs. FFAR4^fl/fl^ + CP). Data are presented as mean ± SD. CP cisplatin

Importantly, TEC-specific ablation of FFAR4 was implemented to identify the effect of renal tubular FFAR4 in AKI induced with cisplatin (Cdh16-Cre+FFAR4^f/f^, referred to as FFAR4^tecKO^, Fig. 11a and Supplementary Fig. 24), while sex- and age-matched FFAR4^f/f^ mice were employed as controls. As presented in Fig. 11b, c, compared with cisplatin FFAR4^f/f^ mice, the BUN and sCr levels were evidently raised in the cisplatin FFAR4^tecKO^ mice. Moreover, the histological analysis indicated that TEC-specific FFAR4 deletion aggravated pathological kidney injury caused by cisplatin (Fig. 11d, e), which was consistent with the results of KIM1 and NGAL mRNA level (Fig. 11f, g). Subsequently, cisplatin-induced FFAR4^tecKO^ mice aggravated senescence phenotypes (confirmed by SA-β-gal positive regions, and the LaminB1, p21, p53, p-Rb/Rb, ɣH2A.X and IL-6 expression) than those of cisplatin-FFAR4^f/f^ group (Fig. 11h, j). Additionally, there were no apparent differences found between FFAR4^tecKO^ and FFAR4^f/f^ groups in senescence phenotype, renal tubular injury, or renal function (Fig. 11b–j). In conclusion, these results indicated that TEC-specific FFAR4 deletion could exacerbate cellular senescence and renal injury in mice with cisplatin-induced AKI.

## Discussion

GPCRs are a large family of proteins involved in numerous biological procedures and have been developed successfully in the pharmaceutical industry for a 20-30% market share of target drugs.^18^ In the current work, we identified that the GPCR member FFAR4 expression was abnormally reduced in renal tubular epithelial cells of the injured kidney. And FFAR4 activation alleviated kidney dysfunction and pathological damage in AKI mice, whereas systemic and TEC-specific knockout of FFAR4 aggravated the severity of the disease. These results emphasize that FFAR4 is a potential target for AKI.

The current research demonstrates for the first time that FFAR4 is a key determinant of cellular senescence both in injured kidneys of AKI mice and TECs, as suggested by the activity of SA-β-gal, the biomarker p53, p21, Lamin B1, ɣH2A.X, phospho-Rb change, and the secretory phenotype IL-6 expression. The regulation of tubular epithelial cell senescence by FFAR4 is a novel finding of this study. Our results examined that the undocumented anti-senescent role of renal tubular FFAR4 is regulated through the aging-associated SirT3 expression via Gq subunit-mediated CaMKKβ/AMPK signaling in cisplatin-treated AKI mice and TCMK-1 cells, this may present a novel pathogenic mechanism of AKI.

Senescence, meaning “old” or “aging”, is a term used to describe irreversible cell-cycle arrest.^11,13^ Cellular senescence can be divided into physiological senescence due to telomere shortening (replicative senescence), and accelerated senescence due to extrinsic stimuli (premature senescence).^11,12^ Accelerated senescence plays important and unexplored roles in several diseases, such as cancer, osteoarticular disorders, and metabolic syndrome.^11^ Currently, cellular senescence is believed to be a crucial pathophysiological change in AKI, and the main determinant of cellular senescence after AKI depends on the degree of damage.^9,10,14,38^ Existing evidence indicates that TECs are implicated in rhabdomyolysis, ischemia-reperfusion injury, and cisplatin-induced cellular senescence in the experimental AKI.^9,39,40^ Nonetheless, no researches have demonstrated senescent cell accumulation in AKI induced by cisplatin. The present study used multiple approaches to confirm the presence of accelerated senescence in injured kidneys and TECs.

Currently, the main markers utilized for the detection of senescence include SA-β-gal, cell cycle-related p53, p16, p21, DNA damage-related γH2A.X, nuclear morphological marker Lamin B1, and the SASP component IL-6.^11–13^ Our present results indicate that the p21 and p53 expression was evidently raised in AKI induced by cisplatin with a decrease in phosphorylated Rb. Notably, p16 is another key senescence biomarker. However, p16 expression did not change in the kidneys of TECs or AKI mice induced by cisplatin (Supplementary Fig. 25). Many studies suggested that extrinsic stimuli could lead to DNA damage which activates p53 as well as its downstream transcriptional target p21 directly, who further induces Rb dephosphorylation and results in cell cycle arrest.^13,41^ In addition to p53-p21 signaling, the p16-Rb pathway is also involved in cellular senescence.^11–13^ There is a view that p21 is activated upon entry into early senescence, while p16 is activated at a later stage, which might partially explain the unchanged p16 expression in the acute phase of cisplatin-induced kidney injury.^11^

SirT is a member of NAD^+^-dependent enzyme family, and mammalian SirT comprises seven members (SirT1-7), which are well-known longevity proteins.^28^ SirT3 is localized in the mitochondria or nucleus and could play regulatory roles in aging, together with aging-associated diseases.^34–36^ Despite the SirT3 mechanism in senescence remains multiplex, the SirT3/ROS/p53 pathway has been widely accepted and investigated.^30,31^ Currently, mitochondria are viewed as a primary source of ROS, and SirT3 could decrease the ROS production by deacetylating superoxide dismutase 2 (SOD2) and further downregulating p53/p21/Rb signaling.^42–44^ The renoprotective effects of SirT3-ROS pathway also have been reported in AKI.^28,35,45,46^ In the current research, we observed that cisplatin decreased the expression of SirT3 in the injured kidneys and TECs, and FFAR4 activation recovered cisplatin-induced reduction of SirT3. Hence, we hypothesized that FFAR4 may facilitate SirT3 expression, thereby improving cellular senescence in AKI induced by cisplatin. The findings of this study supported our hypothesis that silencing SirT3 reversed anti-senescent effect of TUG891, and SirT3 overexpression partially counteracted FFAR4 silencing-mediated exacerbation of tubular cell senescence.

Interestingly, the mechanism of FFAR4 regulates the SirT3 expression. The activation of FFAR4 potentiates Gq-coupled receptor-mediated synthesis of IP3 and intracellular Ca^2+^ release, where CaMKKβ is activated and phosphorylates AMPK.^21,32,33,47^ Recently, growing evidence has validated the regulation of SirT3 by AMPK.^34–36^ In this study, Gq knockdown reversed anti-senescent effect of TUG891, and AMPK inhibitor compound C counteracted TUG891-induced SirT3 upregulation in cisplatin-stimulated TCMK-1 cells. Collectively, our data suggested that FFAR4 upregulated SirT3 expression via Gq subunit-mediated CaMKKβ/AMPK in cisplatin-injured TECs. In parallel, further researches are essential to delineate the mechanisms that various stimuli downregulate FFAR4 expression under AKI conditions.

It is worth emphasizing that there exist opposite research about the relationship between cell senescence and AKI in previous studies. On the one hand, a study on IRI-induced AKI has concluded that cell cycle arrest may act as a protective mechanism after AKI, p21(-/-) mice were more vulnerable to acute renal failure induced with ischemia.^48^ On the other hand, after the above-mentioned study, more and more studies have turned to support the conclusion that cell senescence aggravates AKI. Specifically, the deletion of cell senescence mediator p16 was reported to alleviated cell senescence and rhabdomyolysis (RM)-induced AKI,^49^ and paricalcitol was also reported to attenuate contrast-induced AKI by alleviating senescence and mitophagy.^50^ Besides, restoration of renal function in a rat model of AKI induced with sepsis via suppressing the senescence of renal tubular cells with lipase A4,^51^ and there was also a study reported the renal protection by inhalation of hydrogen-rich aerosols in a mouse model of septic AKI through inhibition of senescence, apoptosis,^52^ etc. Although the previous researches still remain controversial, a recent review has concluded that new evidence suggests that aging helps the advancement of AKI and that inhibiting senescence can facilitate kidney recovery.^53^ Based on our data in cisplatin-, IRI-, and CLP-induced mice models, we also believe that cellular senescence is implicated in facilitating the progress of AKI. In addition, we have set up the rapamycin treatment group as a positive control group of anti-cell senescence, and the experimental data indicated that inhibition of cell senescence by rapamycin could alleviate the kidney damage and dysfunction induced by cisplatin. Consequently, we could speculate inhibiting senescence could mitigate AKI. We suppose that there would be more researches on AKI and cellular senescence and the action of cellular senescence in the occurrence and development of AKI will become more clear.

In conclusion, our study highlights that FFAR4 regulates cellular senescence via AMPK/SirT3 signaling in cisplatin-induced AKI, providing robust evidence that FFAR4 might as a promising target for the treatment of AKI.

## Materials and methods

### Chemicals and antibodies

TUG891 (SML1914) was acquired from Sigma-Aldrich (St. Louis, MO, USA). Cisplatin (D8810) was provided by SolarBio (Beijing, China). Compound C (866405-64-3) was purchased from the MCE (Shanghai, China). Rapamycin (R8140) was provided by SolarBio (Beijing, China). The primary antibodies utilized are presented in Supplementary Table 1. While the secondary antibodies for horseradish peroxidase conjugation were acquired from Thermo Fisher Scientific (Waltham, MA, USA).

### Animals

The protocol of the animal research was reviewed and authorized through the Experimental Animal Ethics Committee of West China Hospital, Sichuan University (2020192A). FFAR4 knockout (KO) mice, Male C57BL/6J mice (Supplementary Fig. 26), and TEC-specific FFAR4 mice (Supplementary Fig. 27) were acquired from Gempharmatech Co. Ltd. (Nanjing, China). All animals were randomly grouped (6 mice per group). The cisplatin group was provided an intraperitoneal injection (i.p.) of body weight (20 mg/kg) of cisplatin, while the control group was provided an equal volume of 0.9% saline. TUG891, an agonist, was administered via gavage at a dose of 35 mg/kg per day for six days, and cisplatin was injected on the 3rd day. Rapamycin was administered intraperitoneally for six days at 2 mg/kg/day in rapamycin group, while cisplatin was injected on the 3rd day. Three days after injecting of cisplatin, the mice were sacrificed. IRI model and CLP model were established as previously described.^54,55^ The animal researches together with the details of the TEC-specific FFAR4 KO mice and FFAR4-KO mice production are concluded in the Supplementary Methods.

### Renal function and histologic examination

Blood samples were taken and next centrifuged at 3000 rpm for 20 min at ambient temperature in order to get serum. The levels of BUN and sCr were determined with an automated biochemical analyzer (Mindray BS-240, Shenzhen, China). Remove half of the kidney, freeze in a liquid nitrogen and keep under a temperature of −80 °C. One-quarter of the kidney was excised and then fixed in 10% formaldehyde (50-00-0, Chron Chemicals, Chengdu, China), dehydrated, embedded into the paraffin and sectioned at a thickness of 4 μm for H&E staining. An additional quarter of the kidney were embedded into OCT compound, which was frozen under a temperature of −80 °C. With AxioCamHRc digital camera (Carl Zeiss, Jena, Germany), kidney sections were observed at 200x and 400x magnification. Renal tubular injury was assessed on a semiquantitative scale of 0–4 as described below: 0, normal; 1, <25% injury; 2, 25–50% injury; 3, 51–75% injury; and 4, >75% injury.^56,57^

### Immunohistochemistry

The paraffin-embedded kidneys were sectioned to a thickness of 4 μm, de-paraffinized, rehydrated, and next antigen-retrieved. Subsequently, these sections were blocked with 2.5% normal goat serum, and inoculated by the primary antibodies anti-p53 (M1312-2, HuaAn Biotechnology, Hangzhou, China) and anti-Ki67 (ET1609-34, HuaAn Biotechnology, Hangzhou, China) diluted 200:1 in PBS under a temperature of 4 °C overnight. Slides were cleaned in PBS 3 times and stained with the VECTASTAIN ABC kit (Vector, Burlingame, CA, USA), which were then visualized under 200x and 400x magnification through utilizing AxioCamHRc digital camera (Carl Zeiss, Jena, Germany) with ZEN 2012 microscopy software (blue version).

### Immunofluorescence

Sections of OCT-embedded kidneys were 4 μm in thickness and subsequently inoculated at room temperature utilizing 5% horse serum for 60 min for blocking the non-specific binding sites. The slides were next inoculated overnight in a humid chamber under a temperature of 4 °C after dilution 1:200 in PBS with primary antibody anti-FFAR4 (ab223512, Abcam, Cambridge, MA, USA) and anti-p21 (ER1906-07, HuaAn Biotechnology, Guangzhou, China). The equivalent secondary antibody (1:500 dilution, 111-025-003, Jackson ImmunoResearch, West Grove, PA, USA) was utilized for 60 min. The proximal tubules were labeled with Fluorescein-labeled Lotus quadrangular lectin (dilution 1:400, FL-1321; Vector Laboratories, CA, USA). Slides were re-cleaned, stained with DAPI (dilution 1:500, D8200, Solarbio, Beijing, China), and sealed through coverslips. Images were gathered at 200x and 400x magnification under an AxioCamHRc digital camera (Carl Zeiss, Jena, Germany) via utilizing the ZEN 2012 microscopy software (blue version).

### RNA-sequencing

Frozen kidney samples from four groups (*n* = 4 per group) were chosen randomly for sequencing. With TRIzol reagent (Invitrogen, Carlsbad, CA, USA), the total RNA of the samples could be extracted, and then the samples were examined for purity, quality, and integrity. Through LC-BIO Bio-Tech Ltd (Hangzhou, China), the construction and sequencing of libraries were conducted. With Illumina NovaSeq 6000 platform, such libraries were subsequently sequenced and paired-end reads with a 2 × 150 bp read length were produced.

### Western blot analysis

Western blot analysis protocols have been previously described.^26^ Immunoblots were observed through Odyssey infrared imaging system. (Fluorescence Chemiluminescence Imaging System, Clinx Science, Shanghai, China) and quantified through utilizing ImageJ software (version 6.0; Wayne Rasband, National Institutes of Health, USA). All of the western blotting assays were performed and repeated 3 times.

### Quantitative real-time PCR analysis

Separation of total RNA, as well as RT-qPCR, was conducted as previously described.^26^ The primers employed for target genes are presented in Supplementary Table 2. The relative expression of genes in comparison with controls was normalized to the expression of GAPDH and calculated through CFX Manager™ Software (Bio-Rad, Hercules, CA, USA).

### Cell culture and treatments

TCMK-1, the tubular epithelial cells in mice were acquired from ATCC institution Shanghai Limai Biological Engineering Co., Ltd (Shanghai, China) and cultivated in MEM/EBSS medium (SH30024.01, Hyclone, Beijing, China) with 10% FBS under 37 °C and a humidified atmosphere of 95% air and 5% CO_2_. PTC were incubated in accordance with the protocol as previously described.^58^ Briefly, PTC were cultured from collagenase-digested kidneys obtained from male C57/BL6J mice (3–4 weeks). The removed kidneys were sliced into 1 mm pieces and transferred to collagenase solution (17100-017, Thermo Fisher Scientific, Waltham, MA, USA) under 37 °C for half an hour of digestion. After that, the supernatant was passed through three nylon sieves (with pore sizes of 40, 70, and 100 μm). The retained proximal tubules on the sieve (40 μm) were resuspended in RPMI 1640 (SH30027.LS, Hyclone, Beijing, China) involving 10% FBS and subsequently centrifuged at 1000 rpm for 10 min. The supernatant was discarded, and 1 ml erythrocyte lysate (R1010, Solarbio, Beijing, China). Next, the solution was centrifuged at 1000 rpm for 5 min. Eventually, discard the supernatant and resuspend it in a suitable amount of medium. RPMI 1640 with 10% FBS, 1% penicillin-streptomycin solution (SV30010, Hyclone, Beijing, China), 1% insulin-transferrin-selenium (abs9463, Absin, Shanghai, China), and epidermal growth factor (20 ng/ml) (RP-10914, Invitrogen, CA, USA) in a humidified atmosphere of 95% air and 5% CO_2_ at 37 °C. Medium was changed in every two days. For cisplatin-induced aging research, TCMK-1 and PTC were incubated in MEM medium supplemented with 2 µg/ml cisplatin for 6 h.^17^ For assessing the treatment effect of TUG891, cells were pre-cultured by TUG891 (10 μM) for 60 min prior to cisplatin treatment for six hours. For checking the effectiveness of AMPK suppression, cells were pre-incubated for 60 min with TUG891 and compound C (10 μM) and subsequently treated by cisplatin for six hours. Details of transfection of SirT3 siRNA, FFAR4 siRNA, negative control (NC) siRNA, Gq siRNA, FFAR4 expression plasmid (pFFAR4), SirT3 (pSirT3) expression plasmid, and blank plasmid (pVector) in TCMK-1 cells are presented in Supplementary Methods.

### Reactive oxygen species detection

With an AxioCamHRc digital camera (Carl Zeiss, Jena, Germany), the detection of ROS in kidneys was carried out, stained in situ through the oxidative fluorescent dye dihydroethidium (DHE) (Sigma-Aldrich, St. Louis, MO, United States). While the AxioCamHRc digital camera (Carl Zeiss, Jena, Germany) and 2′,7′-dichlorofluorescein diacetate were employed for determining the ROS in TCMK-1 cells, in accordance with the guidelines of manufacturer (Beyotime Biotechnology, Shanghai, China).

### SA-β-gal staining

The activity of SA-β-gal was investigated in accordance with the guidelines of the manufacturer utilizing a kit (C0602, Beyotime Biotechnology, Shanghai, China). Images were gathered randomly with an AxioCamHRc digital camera (Carl Zeiss, Jena, Germany).

### Statistical analysis

Random assignment was conducted with the random number table approach. All tests were carried out in triplicate. Data are presented as mean ± SD. Mann-Whitney U test (nonparametric data) or two-tailed Student’s t-test (parametric data) was utilized to analyze the differences in statistics between both groups, while two-way ANOVA (for two experimental parameters) or one-way ANOVA (for a single experimental parameter) was utilized to analyze differences between over two groups, and then Tukey’s multiple comparison assay was implemented. With GraphPad Prism 9.0 (GraphPad Software, San Diego, CA, USA), all the statistical analyses were implemented. *P* was set to *p* < 0.05.

## Supplementary information

SUPPLEMENTAL MATERIAL

## Data Availability

All the data supporting the results of the present study are provided to the corresponding authors upon request.
