# Supplementary material for: FFAR4 improves the senescence of tubular epithelial cells by AMPK/SirT3 signaling in acute kidney injury
Source: Signal Transduct Target Ther. 2022 Nov 30;7:384. doi: 10.1038/s41392-022-01254-x (PMC9712544; doi:10.1038/s41392-022-01254-x)
Supplement: Supplementary file 1 — SUPPLEMENTAL MATERIAL [file 41392_2022_1254_MOESM1_ESM.docx]

Supplementary Materials for

FFAR4 improves the senescence of tubular epithelial cells by AMPK/SirT3 signaling in acute kidney injury

Letian Yang^1^, Bo Wang^1^, Fan Guo, Rongshuang Huang, Yan Liang, Lingzhi Li, Sibei Tao, Ting Yin ^1^, Ping Fu^*^ and Liang Ma^*^

Correspondence to: Liang_m@scu.edu.cn; fupinghx@scu.edu.cn

**This PDF file includes:**

Materials and Methods

Figures. S1 to S27

Tables S1 to S2

Materials and Methods

**Animals**

FFAR4 wild type (WT) and knockout (KO) mice in C57BL/6J background, FFAR4^flox/flox^ (FFAR4^f/f^) and renal tubular epithelial cell-specific (TEC-specific) conditional FFAR4 KO (Cdh16-Cre+FFAR4^f/f^, FFAR4^tecKO^) mice in C57BL/6J background were purchased from GemPharmatech, Nanjing, China. FFAR4 KO target site, sequence details and identification of the genotypes of mice were presented in Supplementary Fig.26. The construction of FFAR4^f/f^ mice is based on CRISPR/Cas9-stimulated homologous recombination. Briefly, exon 2 and exon 3 of the FFAR4 gene were flanked by two LoxP elements. Two heterozygous recombinant embryonic stem cells clones screened by homologous recombination were identified and microinjected into blastocysts from C57BL/6J mice to generate floxed heterozygous mice (FFAR4^flox/+^). FFAR4^flox/+^ mice were then inbred to obtain homozygous FFAR4-floxed mice (FFAR4^f/f^). To generate FFAR4^tecKO^ mice, FFAR4^f/f^ mice were crossed with Cdh16-Cre mice. The genotype of FFAR4^tecKO^ mice was confirmed by PCR assay using specific primers (Supplementary Fig.27). Littermates carried the FFAR4^f/f^ transgene were used as controls.

**Cell transfection with siRNAs**

The mouse FFAR4 siRNA, mouse Gnaq (Gq) siRNA, mouse SirT3 siRNA and mouse negative control (NC) siRNA were purchased from GenePharma (Shanghai, China). TCMK-1 cells transfection with siRNAs was conducted using Lipofectamine 2000 (12566014, Invitrogen, CA, USA) according to the manufacturer’s instructions. The sequences were listed as follows: FFAR4 siRNA, sense 5′-UCACGAAAGCAUCGCGGAATT-3, and antisense 5′-UUCCGCGAUGCUUUCGUGATT-3; Gnaq siRNA, sense 5’- CGACGGGAAUAUCAGUUAUTT -3’ and antisense 5’- AUAACUGAUAUUCCCGUCGTT -3’; SirT3 siRNA, sense 5’- CCCAAUGUCACUCACUACUTT -3’ and antisense 5’- AGUAGUGAGUGACAUUGGGTT-3’; NC siRNA sense 5’-UUCUCCGAACGUGUCACGUTT-3’ and antisense 5’-ACGUGACACGUUCGGAGAATT-3’

**Cell transfection with plasmids**

pCMV-Ffar4(mouse)-6×His-SV40-Neo (P35138), pCMV-SIRT3(mouse)-3×Myc-SV40-Neo (P34699), and pCMV-MCS-3×FLAG-SV40-Neo (P8196) were obtained from MiaoLingPlasmid (Wuhan, China). TCMK-1 cells transfection with plasmids was conducted using Lipofectamine 2000 (12566014, Invitrogen, CA, USA) according to the manufacturer’s instructions.


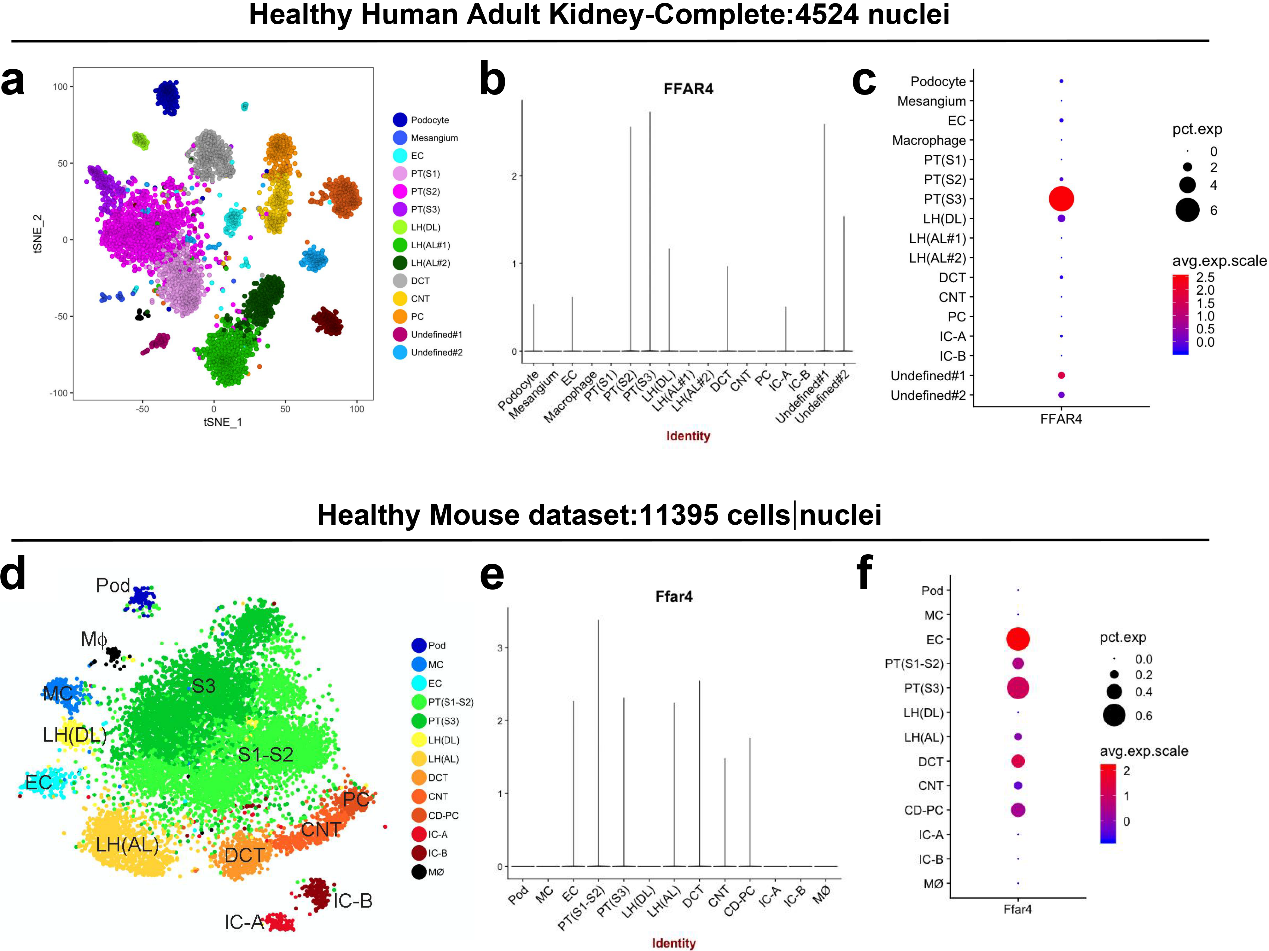


**Figure. S1. The expression and characteristics of FFAR4 in healthy human adult and healthy mouse kidney single cells.** All open data were from single-cell RNA sequencing database (http://humphreyslab.com/SingleCell/). **a-c** Collecting 4524 cells from the healthy adult human kidney, tSNE analysis presented that FFAR4 was a few scattered within the kidney cells. **d-f** Collecting 11395 cells from the healthy mouse kidney, tSNE analysis presented that FFAR4 was a few scattered within the kidney cells. EC, endothelial cells; PT(S1), S1 segment of proximal tubule; PT(S2), S2 segment of proximal tubule; PT(S3), S3 segment of proximal tubule; PT(S1, S2), S1,S2 segment of proximal tubule; LH(DL), loop of Henle descending loop; LH(AL), loop of Henle ascending loop; DCT, distal convoluted tubule; CNT, connecting tubule; Pod, podocyte; MC, mesangial cell; EC, endothelial cell; CD: PC, collecting duct-principal cells; IC-A, intercalated cell type A; IC-B, intercalated cell type B; M.Φ, macrophage.


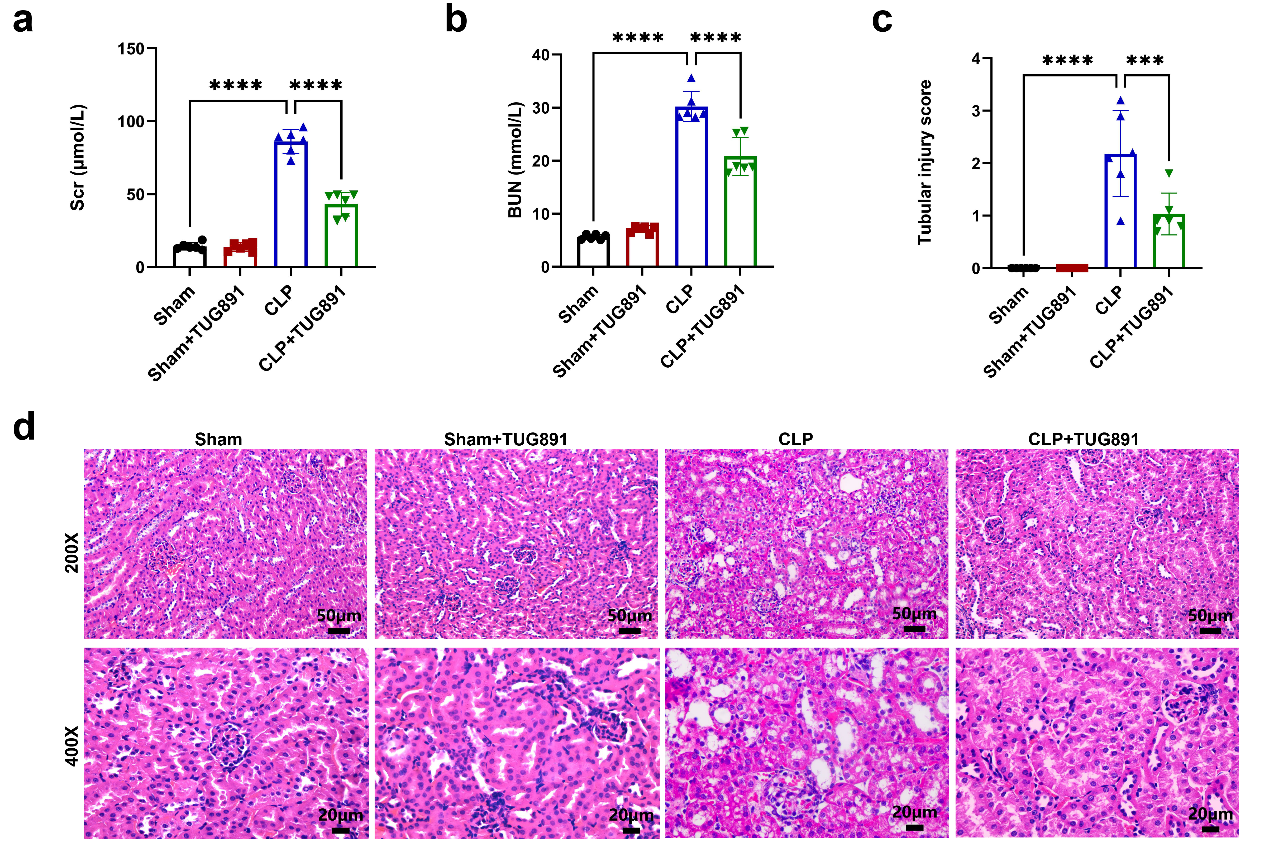


**Figure.S2. Activation of FFAR4 by agonist TUG891 alleviated CLP-induced AKI in mice. a** The sCr level in different groups of mice (n=6). **b** The BUN level in different groups of mice (n=6). **c** Tubular injury scores of kidney tissues (n=6). **d** Representative images of H&E staining (200×, scale bar = 50 μm; 400×, scale bar = 20 μm). Data are presented as mean ± SD. CLP, cecal ligation/perforation. ****P < 0.0001, ***P<0.001


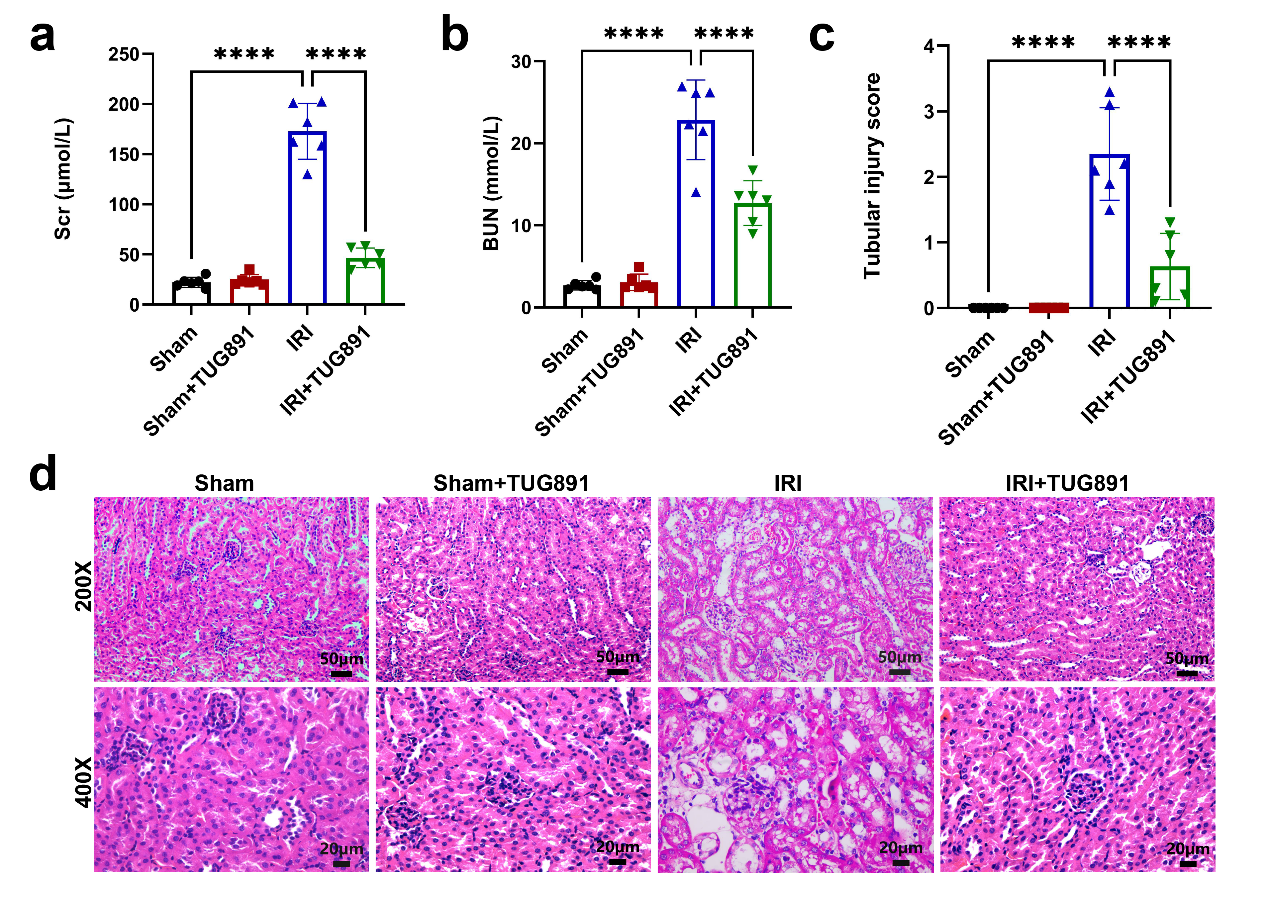


Figure.S3. Activation of FFAR4 by agonist TUG891 alleviated IRI-induced AKI in mice. a The sCr level in different groups of mice (n=6). b The BUN level in different groups of mice (n=6). c Tubular injury scores of kidney tissues (n=6). d Representative images of H&E staining (200×, scale bar = 50 μm; 400×, scale bar = 20 μm). Data are presented as mean ± SD. IRI, ischemia/reperfusion injury. ****P < 0.0001, ***P<0.001


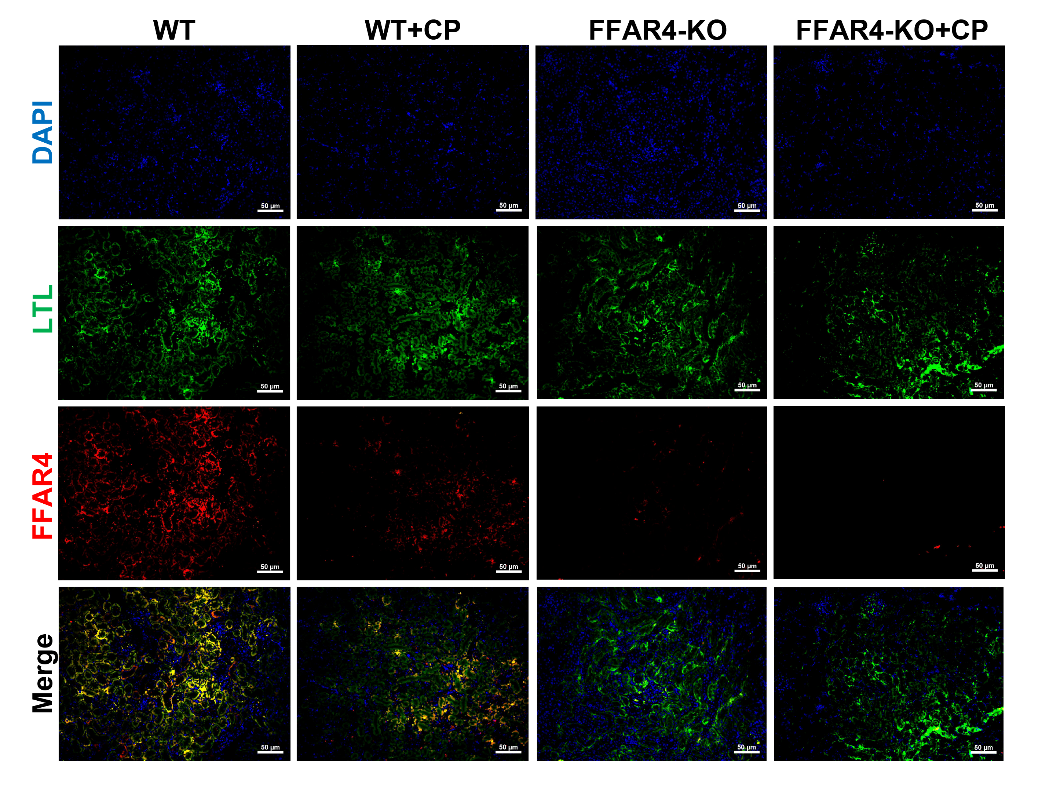


**Figure.S4. FFAR4 deficiency aggravated CP-induced AKI in mice.** Immunofluorescence of FFAR4 (red) in proximal tubules (LTL, green) in kidney sections (200×, scale bar = 50 μm). CP, cisplatin.


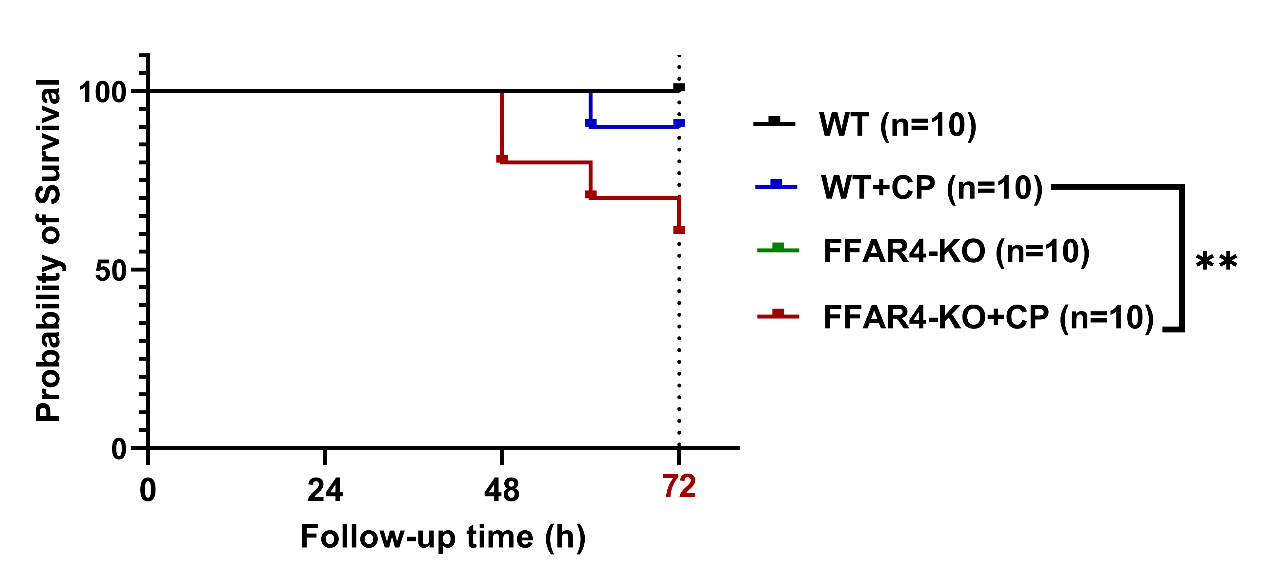


**Figure.S5. FFAR4 deficiency was associated with a worse overall survival of CP-induced AKI mice.** The survival analysis of cisplatin-treated FFAR4-KO mice. CP: cisplatin.


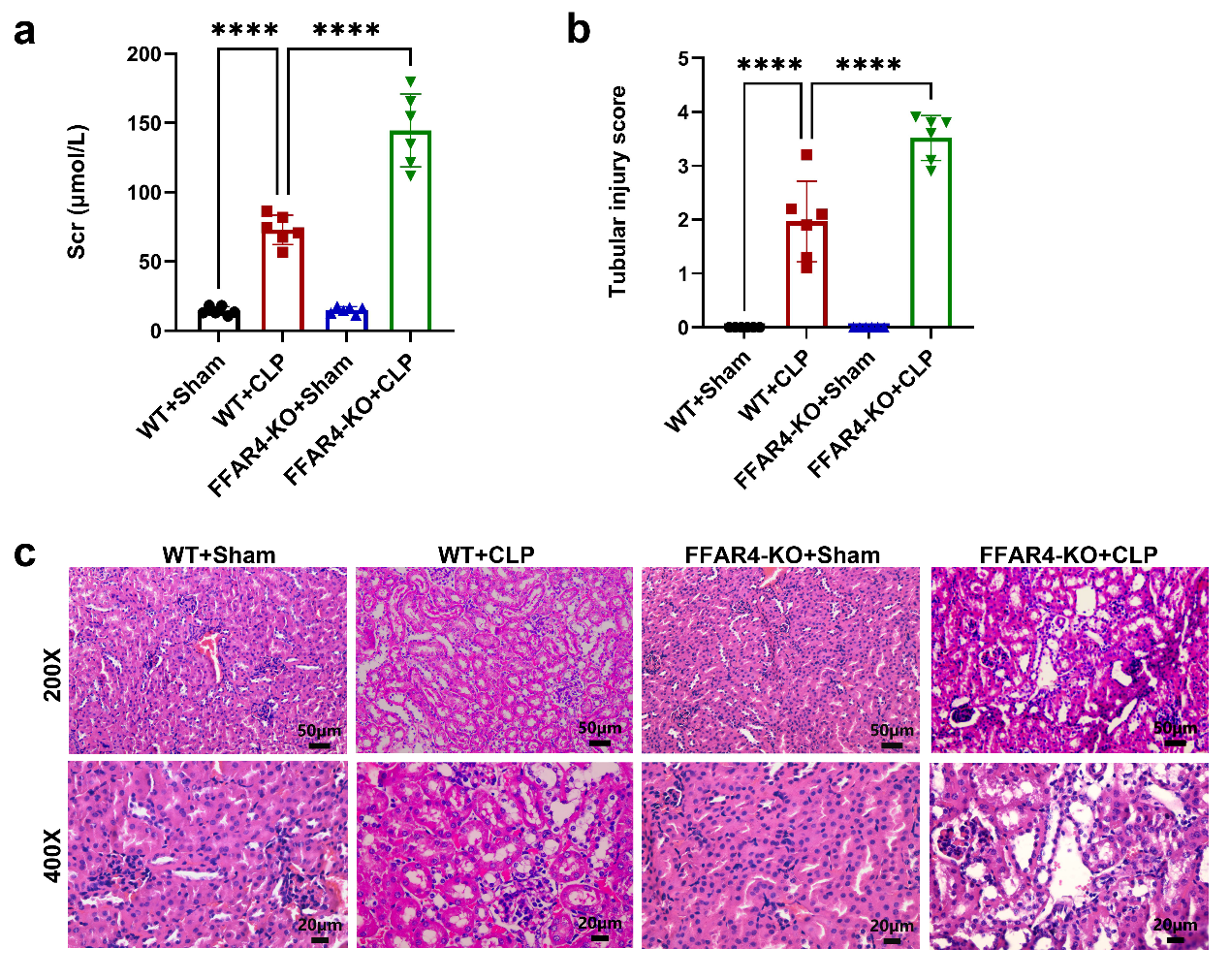


**Figure.S6. FFAR4 deficiency aggravated CLP-induced AKI in mice. a** The sCr level in different groups of mice (n=6). **b** Tubular injury scores of kidney tissues (n=6). **c** Representative images of H&E staining (200×, scale bar = 50 μm; 400×, scale bar = 20 μm). Data are presented as mean ± SD. CLP, cecal ligation/perforation. ****P < 0.0001.


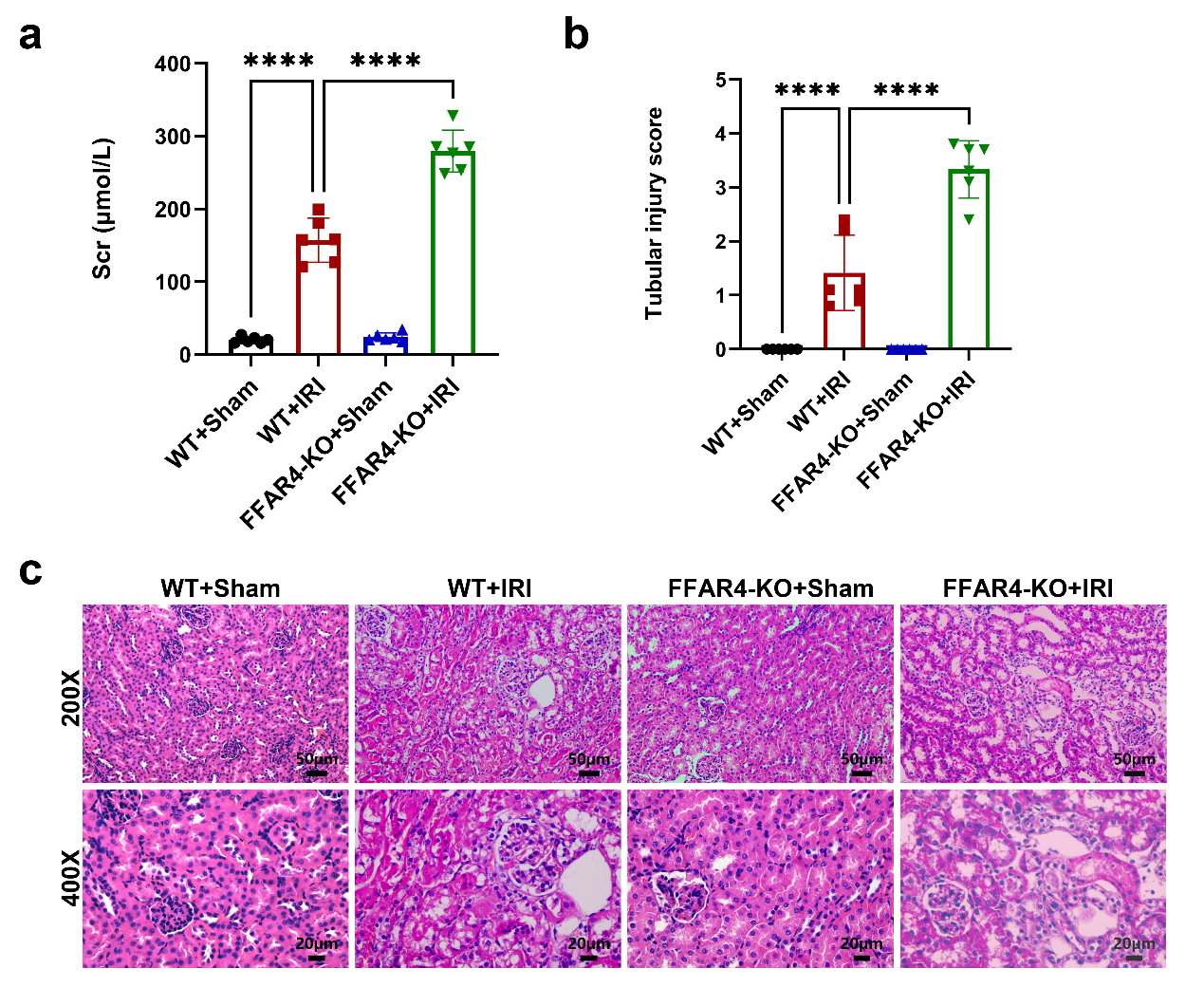


**Figure.S7. FFAR4 deficiency aggravated IRI-induced AKI in mice. a** The sCr level in different groups of mice (n=6). **b** Tubular injury scores of kidney tissues (n=6). **c** Representative images of H&E staining (200×, scale bar = 50 μm; 400×, scale bar = 20 μm). Data are presented as mean ± SD. IRI, ischemia/reperfusion injury. ****P < 0.0001, ***P<0.001


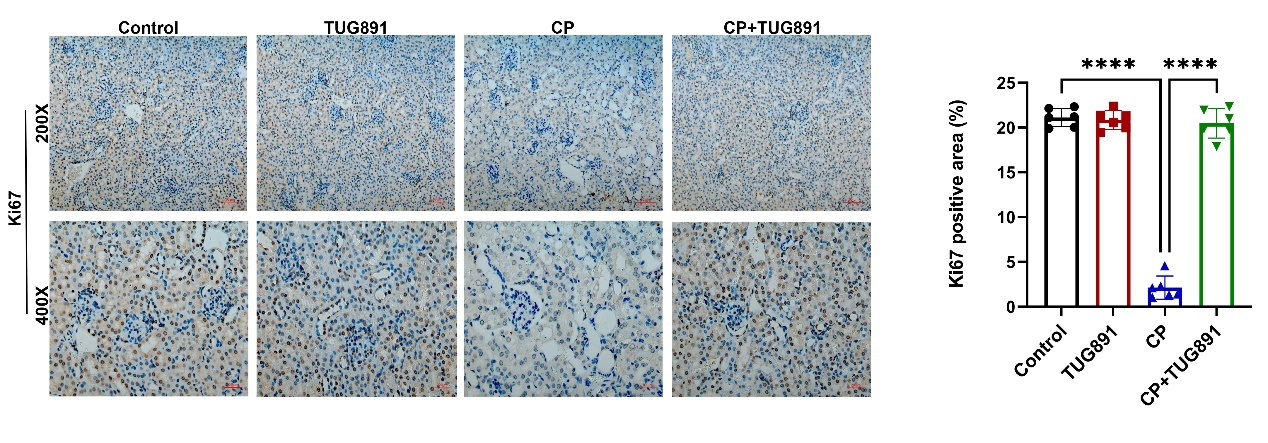


**Figure.S8. TUG891 improved renal cellular senescence in CP-induced AKI mice.** Representative images and quantitative analysis of immunochemistry staining of Ki67 in kidney tissues (200×, scale bar = 50 μm; 400×, scale bar = 20 μm) (n = 6) (****P<0.0001). Data are presented as mean ± SD. CP, cisplatin.


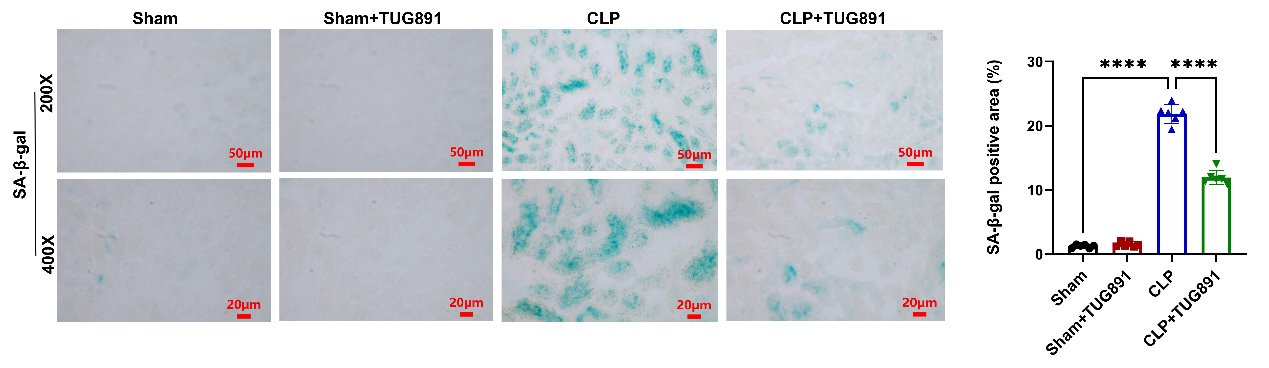


**Figure.S9. TUG891 improved renal cellular senescence of CLP-induced AKI mice.** Representative micrographs and quantitative analysis of SA-β-gal staining of kidney sections (200×, scale bar = 50 μm; 400×, scale bar = 20 μm) (n=6). Data are presented as mean ± SD. CLP, cecal ligation/perforation. ****P < 0.0001.


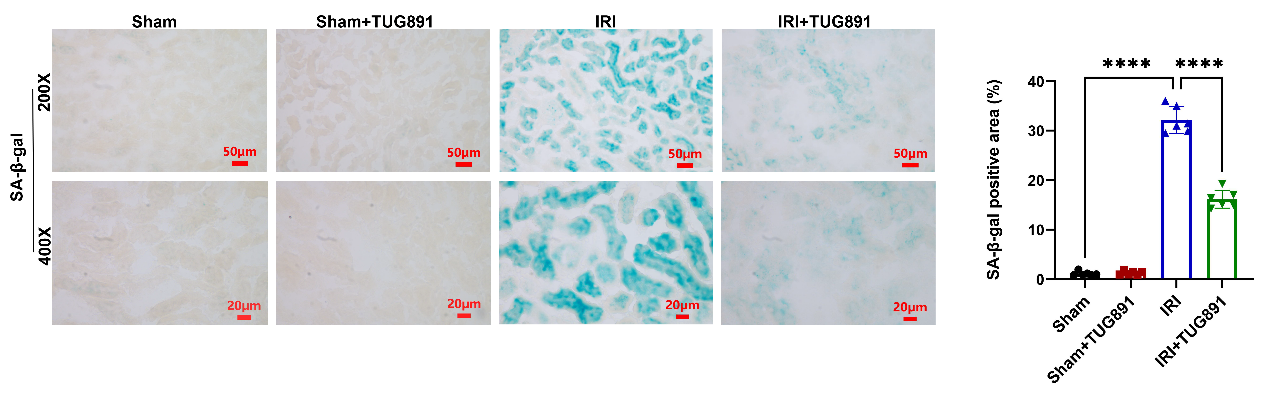


**Figure.S10. TUG891 improved renal cellular senescence of IRI-induced AKI mice.** Representative micrographs and quantitative analysis of SA-β-gal staining of kidney sections (200×, scale bar = 50 μm; 400×, scale bar = 20 μm) (n=6). Data are presented as mean ± SD. IRI, ischemia/reperfusion injury. ****P < 0.0001.


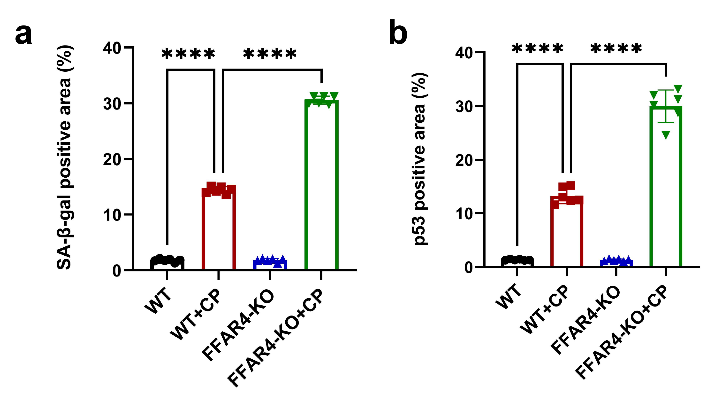


**Figure.S11. FFAR4 deficiency aggravated cellular senescence in CP-induced AKI mice.** **a** Quantitative analysis of SA-β-gal staining of kidney sections (n=6) (Fig.4c). **b** Quantitative analysis of immunochemistry staining of p53 of kidney sections (n=6) (Fig.4d). Data are presented as mean ± SD. CP, cisplatin. ****P < 0.0001.


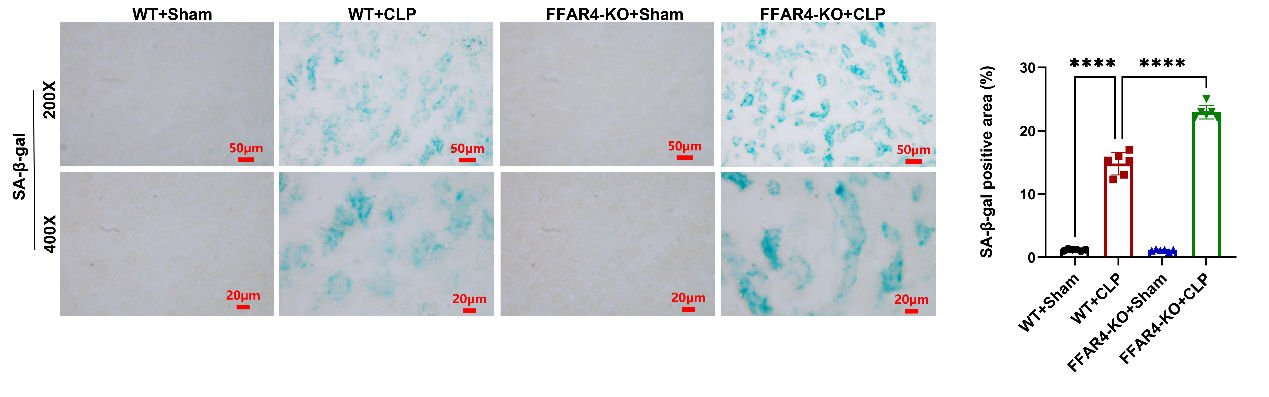


**Figure.S12. FFAR4 deficiency aggravated cellular senescence in CLP-induced AKI mice.** Representative micrographs and quantitative analysis of SA-β-gal staining of kidney sections (200×, scale bar = 50 μm; 400×, scale bar = 20 μm) (n=6). Data are presented as mean ± SD. CLP, cecal ligation/perforation. ****P < 0.0001.


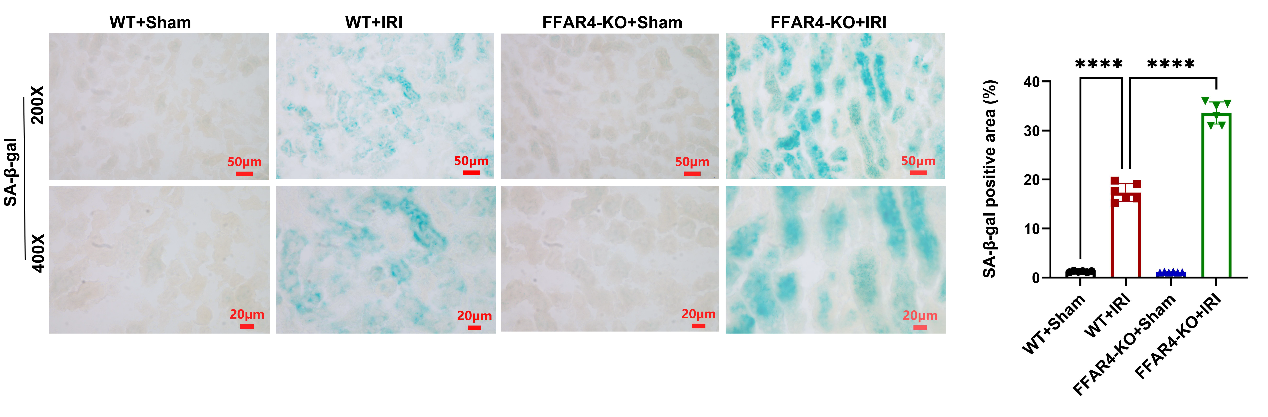


**Figure. S13. FFAR4 deficiency aggravated cellular senescence in IRI-induced AKI mice.** Representative micrographs and quantitative analysis of SA-β-gal staining of kidney sections (200×, scale bar = 50 μm; 400×, scale bar = 20 μm) (n=6). Data are presented as mean ± SD. IRI, ischemia/reperfusion injury. ****P < 0.0001.


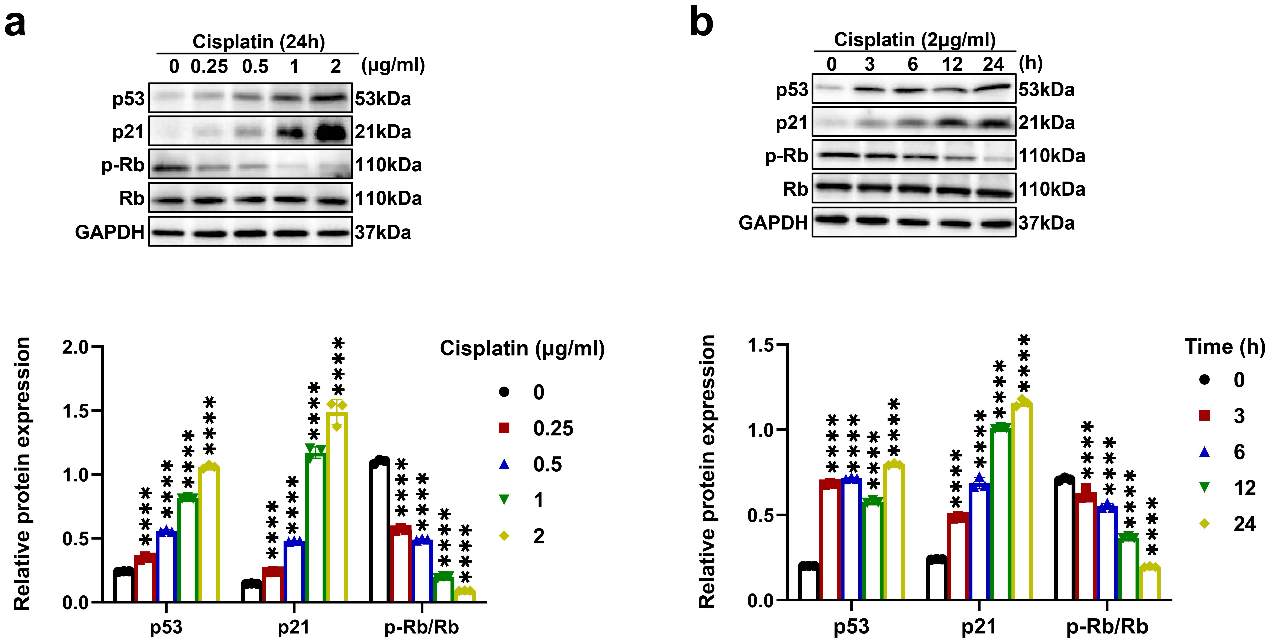


**Figure. S14.** **The effects of concentration and timing of cisplatin-induced cellular senescence in TCMK-1 cells.** Protein expression of p53, p21 and p-Rb/Rb ratio in TCMK-1 cells detected by western blotting and quantified by densitometry (n=3) (a ****P < 0.0001, vs 0 g/ml; b ****P < 0.0001, vs 0 h).


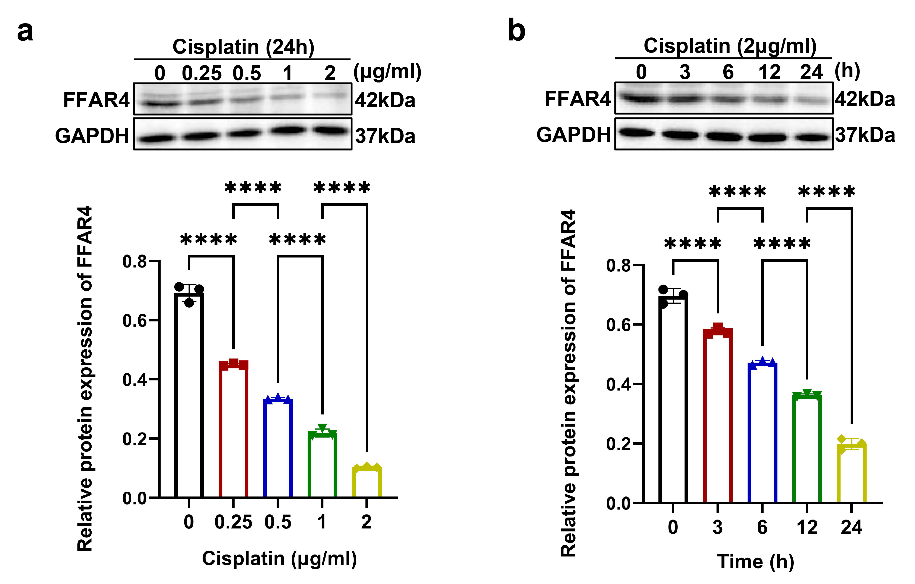


**Figure. S15. The effects of concentration and timing of cisplatin-induced decrease of FFAR4 expressions in TCMK-1 cells.** Protein expression of FFAR4 in TCMK-1 cells detected by western blotting and quantified by densitometry (n=3) (****P < 0.0001).


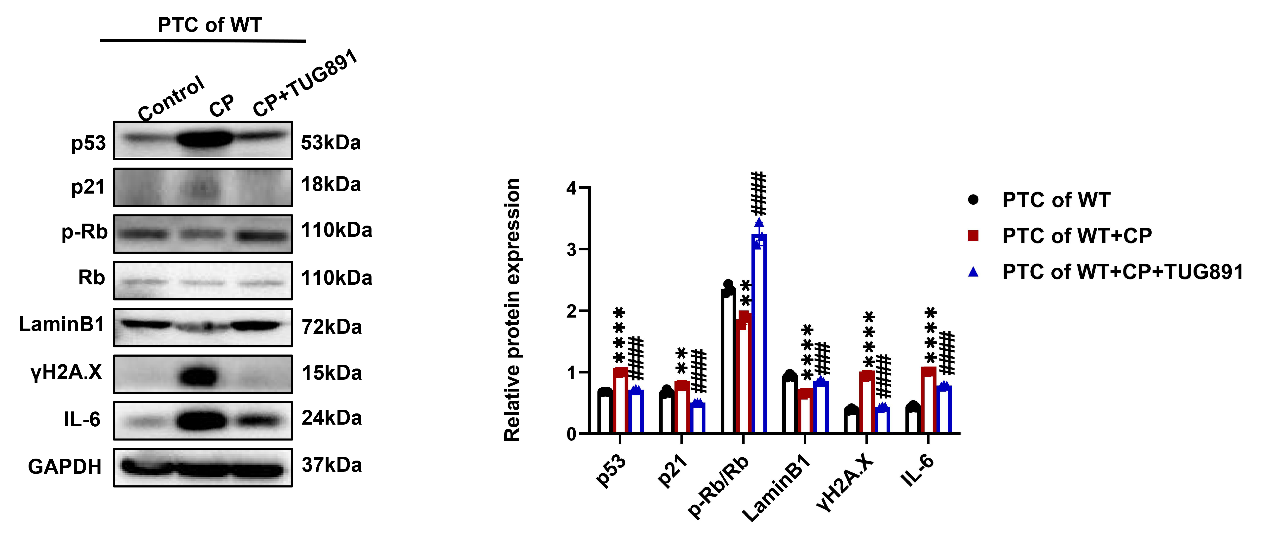


**Figure. S16. TUG891 improved cisplatin-induced cellular senescence in PTCs.** Protein expression of p53, p21, p-Rb/Rb, LaminB1, ɣH2A.X, and IL-6 was detected by western blotting and quantified by densitometry (n=3). Data are presented as mean ± SD. CP, cisplatin. ****P<0.0001, **P<0.01, PTC of WT+CP vs PTC of WT; ####P<0.0001, ###P<0.001, PTC of WT+CP+TUG891 vs PTC of WT+CP.


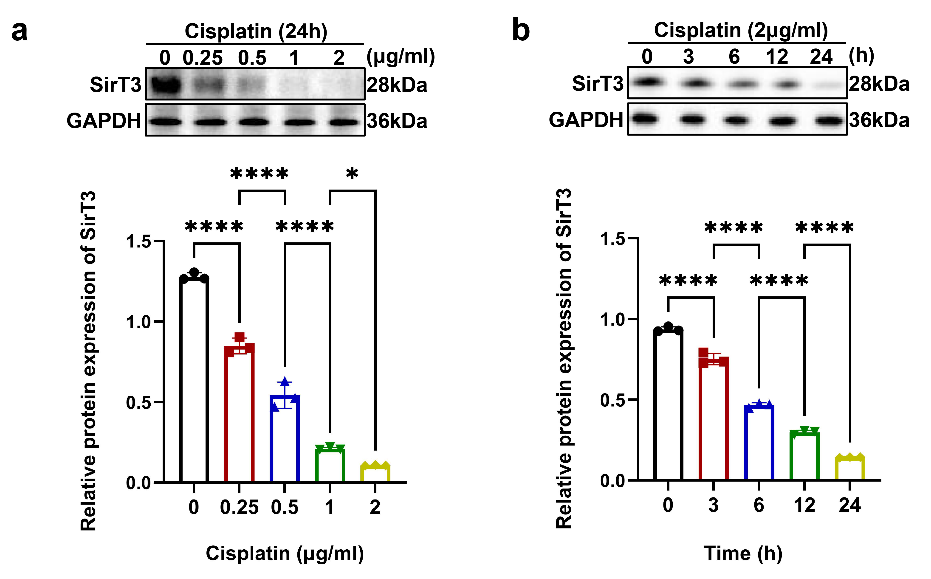


**Figure. S17.** **The effects of concentration and timing of cisplatin-induced decrease of SirT3 expressions in TCMK-1 cells.** Protein expression of SirT3 in TCMK-1 cells detected by western blotting and quantified by densitometry (n=3). Data are presented as mean ± SD. ****P < 0.0001, *P < 0.1.


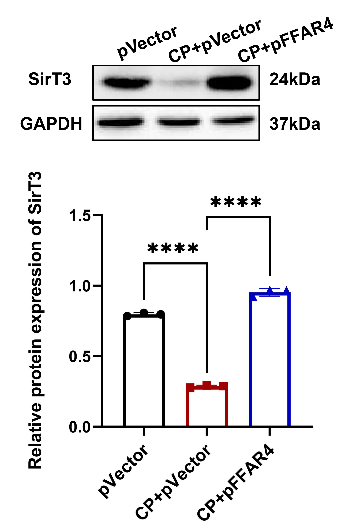


**Figure. S18.** **FFAR4 overexpression reversed the decrease of SirT3 in cisplatin-stimulated TCMK-1 cells.** Protein expression of SirT3 in TCMK-1 cells detected by western blotting and quantified by densitometry (n=3). Data are presented as mean ± SD. CP, cisplatin. ****P < 0.0001.


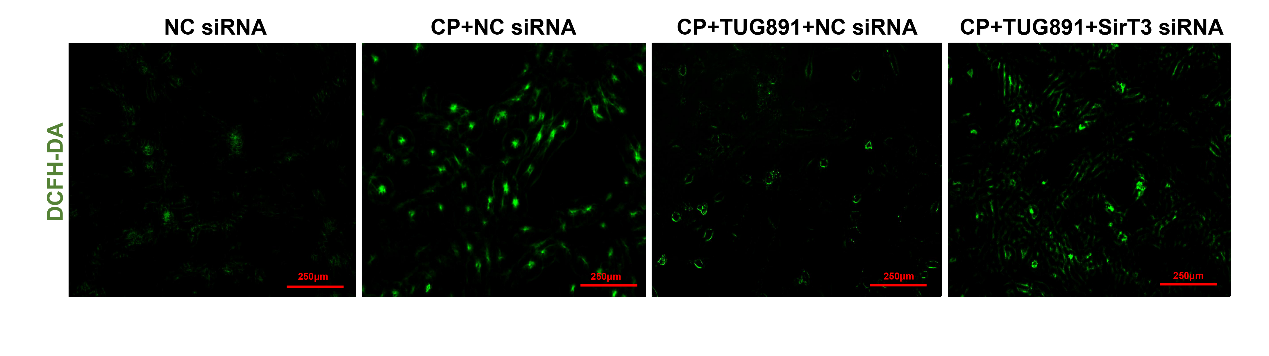


**Figure. S19.** **TUG891 downregulated ROS level via SirT3 pathway in cisplatin-stimulated TCMK-1 cells.** The ROS production in TCMK-1 cells assessed by DCFH-DA staining (100×, scale bar = 250 μm). CP, cisplatin.


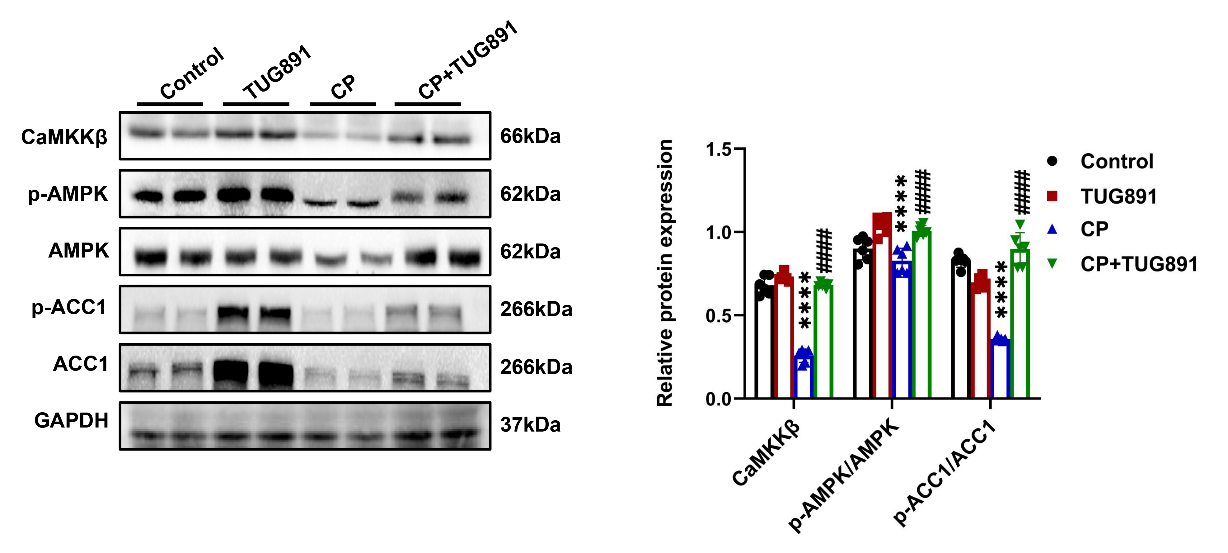


**Figure. S20.** **TUG891 upregulated Sirt3 expression via CaMKKβ/AMPK signaling in cisplatin-induced AKI mice.** Protein expression of CaMKKβ, p-AMPK/AMPK ratio, and p-ACC1/ACC1 in kidney tissues detected by western blotting and quantified by densitometry (n=3). Data are presented as mean ± SD. CP, cisplatin. ****P<0.0001, CP vs Control; ####P<0.0001, CP+TUG891 vs CP.


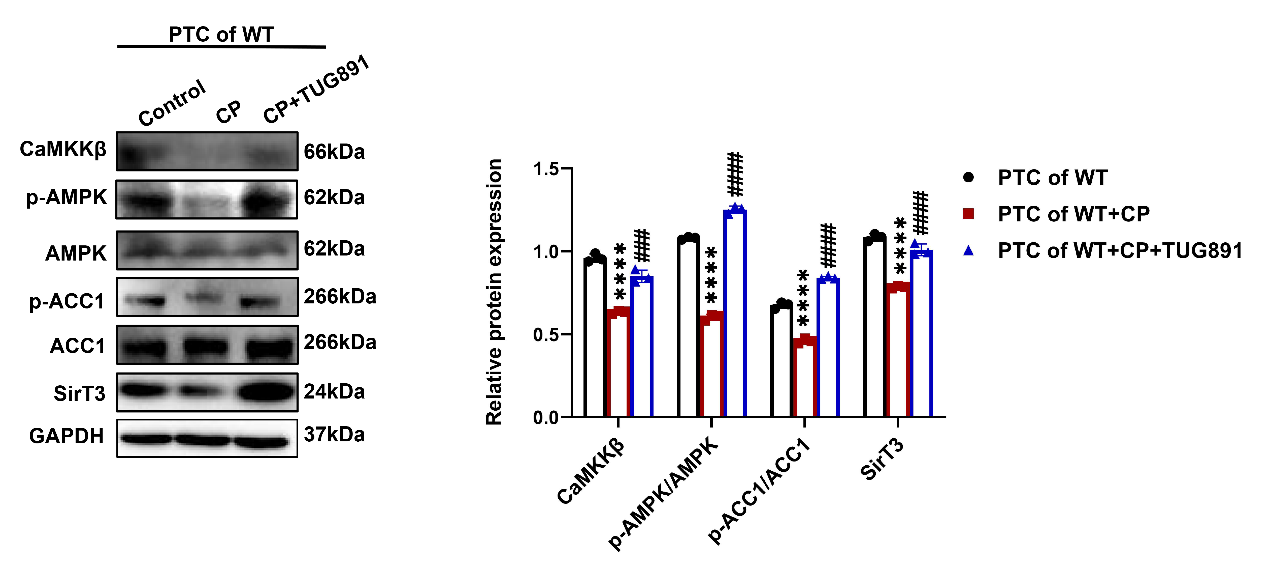


**Figure. S21.** **TUG891 upregulated Sirt3 expression via CaMKKβ/AMPK signaling in PTCs.** Protein expression of CaMKKβ, p-AMPK/AMPK ratio, p-ACC1/ACC1, and SirT3 was detected by western blotting and quantified by densitometry (n=3). Data are presented as mean ± SD. CP, cisplatin. ****P<0.0001, PTC of WT+CP vs PTC of WT; ####P<0.0001, ###P<0.001, PTC of WT+CP+TUG891 vs PTC of WT+CP.


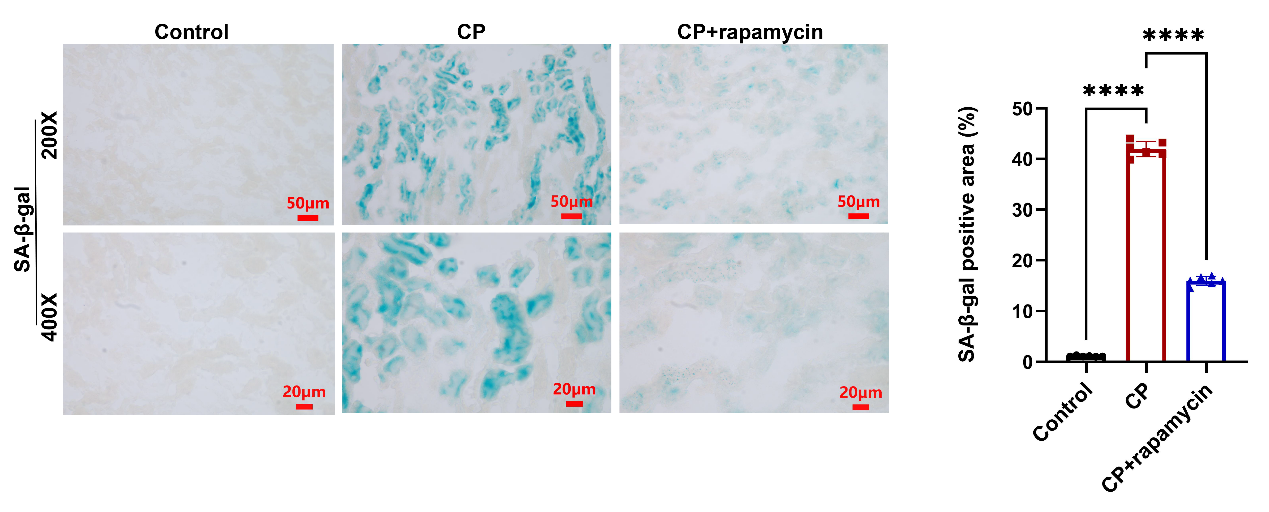


**Figure. S22.** **Rapamycin improved cellular senescence in CP-induced AKI mice.** Representative micrographs and quantitative analysis of SA-β-gal staining of kidney sections (200×, scale bar = 50 μm; 400×, scale bar = 20 μm) (n=6). Data are presented as mean ± SD. CP, cisplatin. ****P<0.0001.


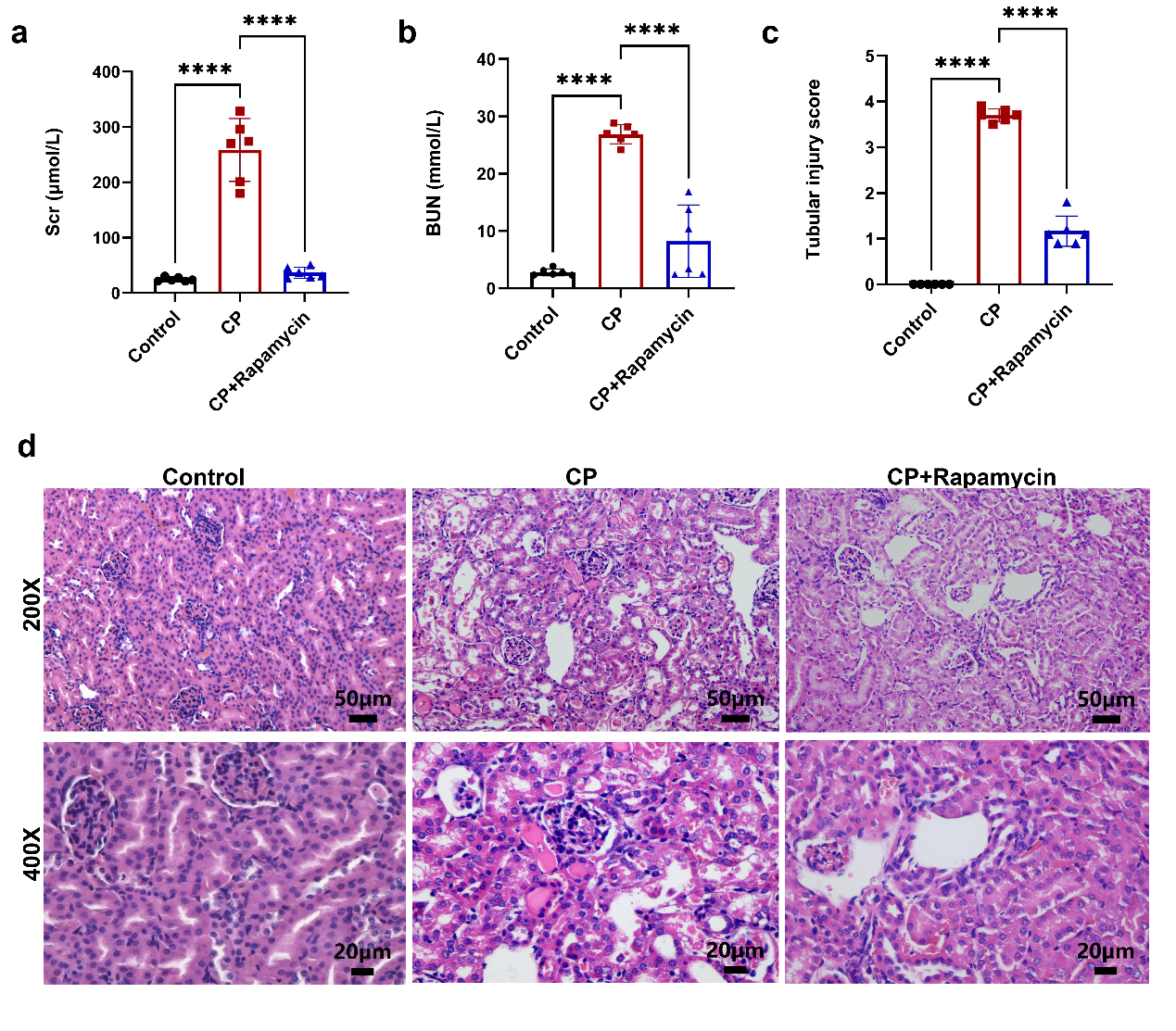


**Figure. S23.** **Rapamycin alleviated CP-induced AKI in mice. a** The sCr level in different groups of mice (n=6). **b** The BUN level in different groups of mice (n=6). **c** Tubular injury scores of kidney tissues (n=6). **d** Representative images of H&E staining (200×, scale bar = 50 μm; 400×, scale bar = 20 μm). Data are presented as mean ± SD. CP, cisplatin. ****P < 0.0001.


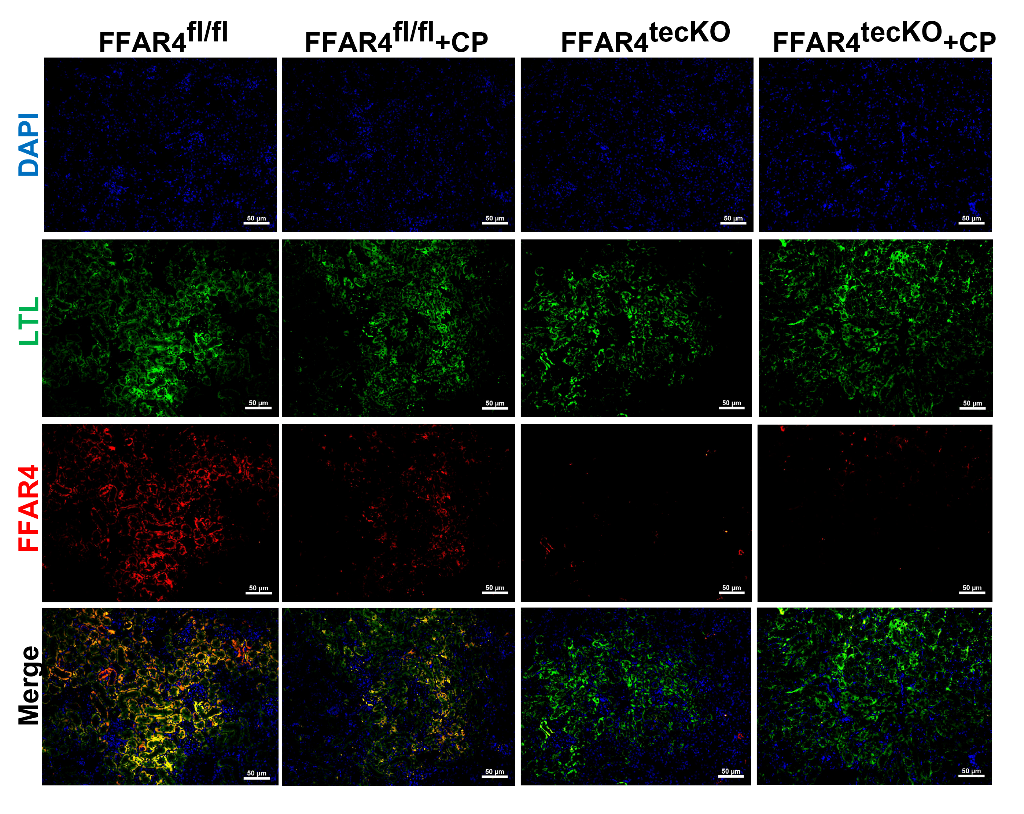


**Figure. S24.** **Tubular epithelial cell-specific deletion of FFAR4 aggravated kidney damage and cellular senescence in cisplatin-induced AKI mice.** Immunofluorescence of FFAR4 (red) in proximal tubules (LTL, green) in kidney sections (200×, scale bar = 50 μm). CP, cisplatin.


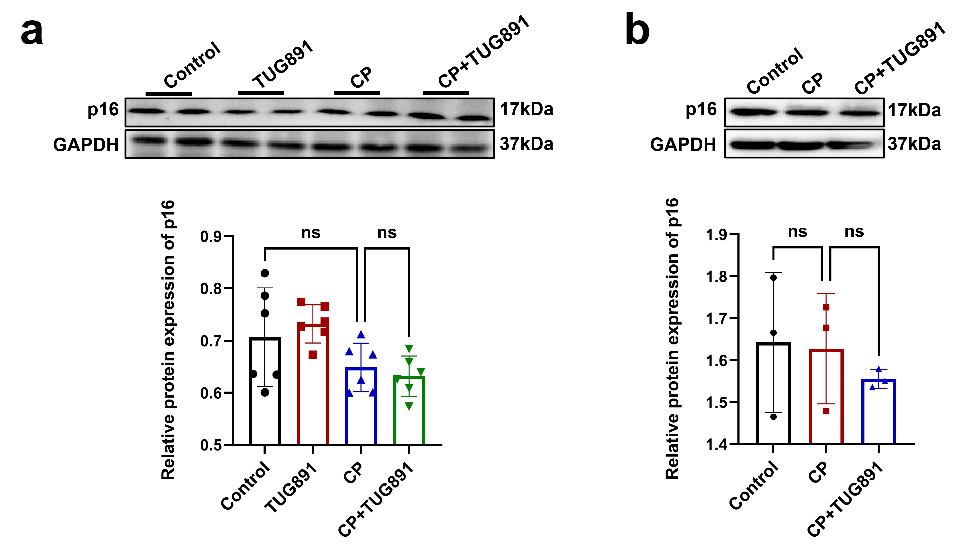


**Figure. S25.** **p16 protein levels did not change in kidneys of cisplatin-induced AKI mice or cisplatin-stimulated TCMK-1 cells.** a Protein expression of p16 in kidney tissues detected by western blotting and quantified by densitometry (n=6). b Protein expression of p16 in TCMK-1 cells detected by western blotting and quantified by densitometry (n=3). Data are presented as mean ± SD. CP, cisplatin. ns, no significant.


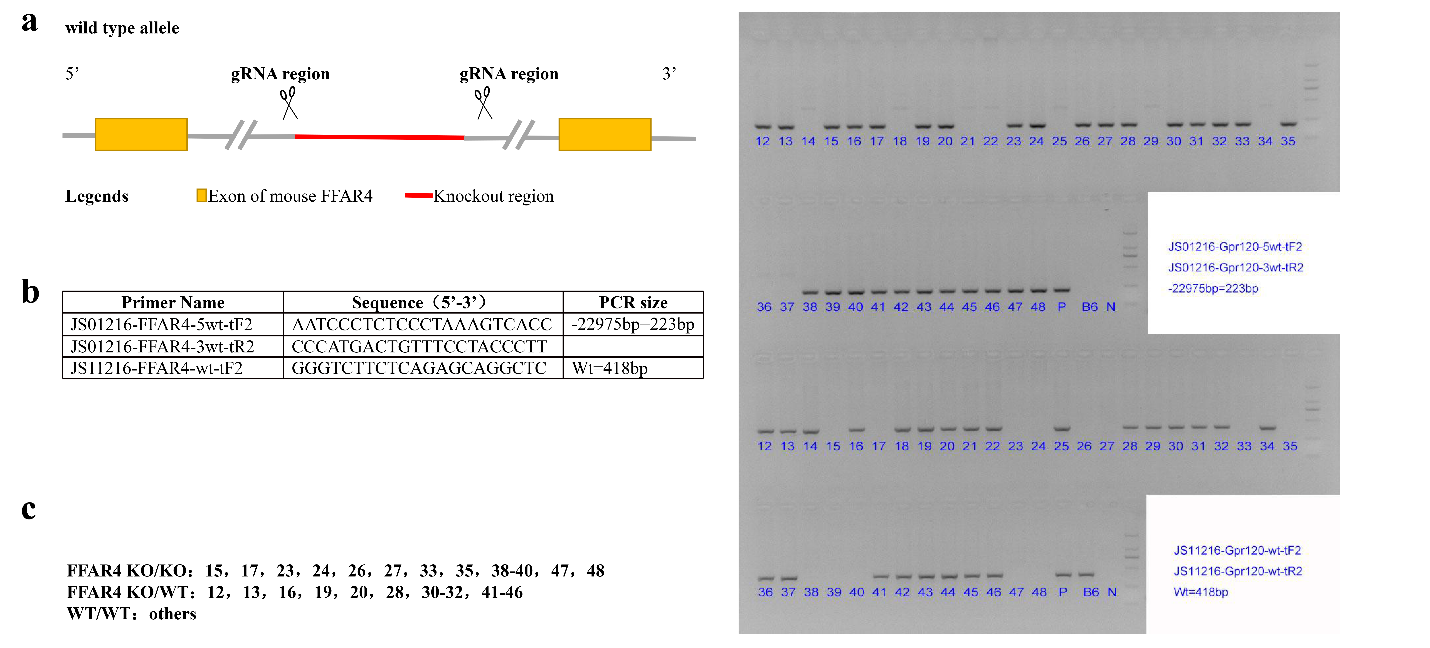


**Figure. S26.** **Generation of FFAR4-KO mice. a** Schematic of FFAR4-KO mice generation. **b, c** Identification of the genotype of FFAR4-KO mice by PCR assay.


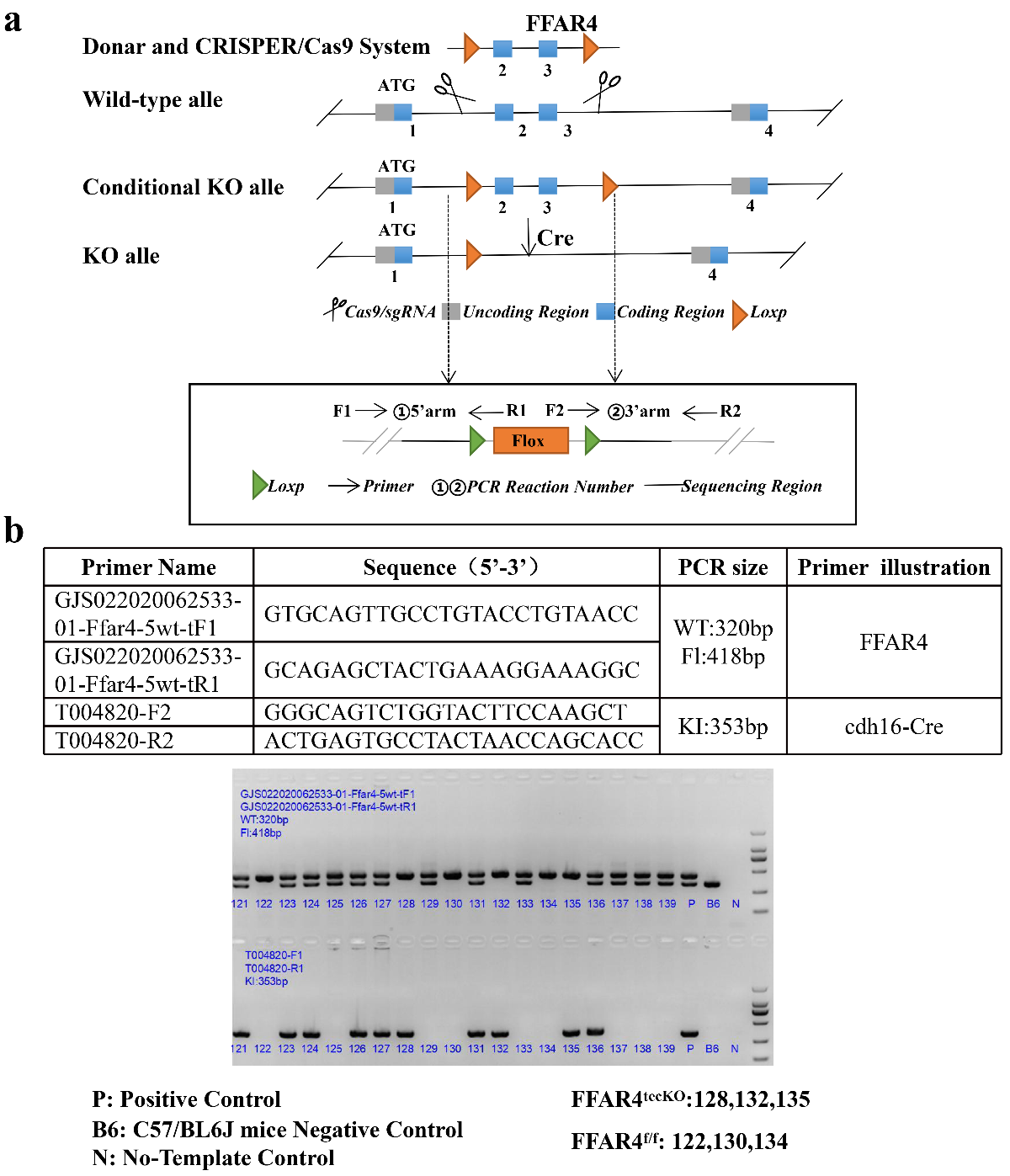


**Figure. S27.** **Generation of renal tubular epithelial cell-specific (TEC-specific) FFAR4 KO mice. a** Schematic of FFAR4^flox/flox^ (FFAR4^f/f^) mice generation by CRISPR/Cas9-stimulated homologous recombination and design strategy of TEC-specific FFAR4 KO (FFAR4^tecKO^) mice. **b** Identification of the genotype of FFAR4^f/f^ mice and FFAR4^tecKO^ (Cdh16-Cre+ FFAR4^f/f^) mice by PCR assay.

**Table S1. The list of primary antibodies.**

| **Name** | **Company** | **Catalog Number** |
| --- | --- | --- |
| Anti-P53 [C8-A11] | HuaAn Biotechnology, Hangzhou, China | M1312-2 |
| Anti-CDKN1A/P21 | HuaAn Biotechnology, Hangzhou, China | HA500156 |
| Anti-CDKN1A/P21 | HuaAn Biotechnology, Hangzhou, China | ER1906-07 |
| Anti-CDKN2A/p16INK4a | HuaAn Biotechnology, Hangzhou, China | ER2001-30 |
| Anti-Ki67 [ST50-01] | HuaAn Biotechnology, Hangzhou, China | ET1609-34 |
| Anti-Phospho-Histone H2A.X (Ser139) | Cell Signaling Technology, MA, USA | 2577 |
| Anti-IL-1β | Cell Signaling Technology, MA, USA | 12242S |
| Anti-IL-6 | HuaAn Biotechnology, Hangzhou, China | EM170414 |
| Anti-TNF-α | Affinity Biosciences, Changzhou, China | AF7014 |
| Anti-Rb (D20) | Cell Signaling Technology, MA, USA | 9313 |
| Anti-Phospho-Rb (Ser807/811) (D20B12) | Cell Signaling Technology, MA, USA | 8516 |
| Anti-LaminB1 (D9V6H) | Cell Signaling Technology, MA, USA | 13435 |
| Anti-FFAR4 | Abcam, MA, USA | ab223512 |
| Anti-FFAR4 | Santa Cruz Biotechnology, CA, USA | sc-390752 |
| Anti-FFAR4 | Novus, CO, USA | NBP1-00858 |
| Anti-SirT3 | Cell Signaling Technology, MA, USA | 5490 |
| Anti-AMPK | Cell Signaling Technology, MA, USA | 2532 |
| Anti-Phospho-AMPK (Thr172) (40H9) | Cell Signaling Technology, MA, USA | 2535 |
| Anti-CaMKKβ | Santa Cruz Biotechnology, CA, USA | sc-517319 |
| Anti-ACC1 | HuaAn Biotechnology, Hangzhou, China | ET1609-77 |
| Anti-Phospho-ACC1 (Ser79) (D7D11) | Cell Signaling Technology, MA, USA | 11818 |
| Anti-GAPDH | Zenbioscience, Chengdu, China | 200306-7E4 |

**Table S2. The list of primer sequences.**

| **Mouse Gene** | **Sequence** |
| --- | --- |
| F-FFAR4 | ACCAAGTCAATCGCACCCAC |
| R-FFAR4 | GTGAGACGACAAAGATGAGCC |
| F-Gnaq | GGTCGGGCTACTCTGACGA |
| R-Gnaq | ACTTGTATGGGATCTTGAGCGT |
| F-SirT3 | ATCCCGGACTTCAGATCCCC |
| R-SirT3 | CAACATGAAAAAGGGCTTGGG |
| F-cdkn1a | CCTGGTGATGTCCGACCTG |
| R-cdkn1a | CCATGAGCGCATCGCAATC |
| F-cdkn2a | CGCAGGTTCTTGGTCACTGT |
| R-cdkn2a | TGTTCACGAAAGCCAGAGCG |
| F-NGAL | GCAGGTGGTACGTTGTGGG |
| R-NGAL | CTCTTGTAGCTCATAGATGGTGC |
| F-KIM1 | ACATATCGTGGAATCACAACGAC |
| R-KIM1 | ACTGCTCTTCTGATAGGTGACA |
| F-IL-6 | ACAACCACGGCCTTCCCTACTT |
| R-IL-6 | CACGATTTCCCAGAGAACATGTG |
| F-IL-1β | TGGGCCTCAAAGGAAAGAAT |
| R-IL-1β | CAGGCTTGTGCTCTGCTTGT |
| F-TNF-α | ACCCTCACACTCAGATCATCTTC |
| R-TNF-α | TGGTGGTTTGCTACGACGT |
| F-IL-8 | CCTACTTCAGCATCCTCTACTGG |
| R-IL-8 | AGGGTTTCTTGAGAAGGGGAC |
| F-GAPDH | GTATGACTCCACTCACGGCAAA |
| R-GAPDH | GGTCTCGCTCCTGGAAGATG |
